# Metabolic Engineering of *Saccharomyces cerevisiae* for Production of Canthaxanthin, Zeaxanthin, and Astaxanthin

**DOI:** 10.3390/jof10060433

**Published:** 2024-06-18

**Authors:** Peerada Promdonkoy, Akaraphol Watcharawipas, Suriyaporn Bubphasawan, Kitisak Sansatchanon, Nattida Suwanakitti, Kanokarn Kocharin, Weerawat Runguphan

**Affiliations:** 1 National Center for Genetic Engineering and Biotechnology, 113 Thailand Science Park, Paholyothin Road, Klong 1, Klong Luang, Pathum Thani 12120, Thailand; peerada.prom@biotec.or.th (P.P.); suriyaporn.bub@biotec.or.th (S.B.); kitisak.san@biotec.or.th (K.S.); nattida.suw@biotec.or.th (N.S.); kanokarn.koc@biotec.or.th (K.K.); 2 Department of Microbiology, Faculty of Science, Mahidol University, 272 Rama VI Road, Ratchathewi, Bangkok 10400, Thailand; akaraphol.wat@mahidol.ac.th

**Keywords:** carotenoids, metabolic engineering, biorefinery, yeast, *Saccharomyces cerevisiae*

## Abstract

The sustainable production of natural compounds is increasingly important in today’s industrial landscape. This study investigates the metabolic engineering of *Saccharomyces cerevisiae* for the efficient biosynthesis of valuable carotenoids: canthaxanthin, zeaxanthin, and astaxanthin. Utilizing a tailored parental yeast strain, Sp_Bc, we optimized the carotenoid pathway by screening and identifying CrtW and CrtZ enzymatic variants. The CrtW variant from *Bradyrhizobium* sp. achieved a canthaxanthin titer of 425.1 ± 69.1 µg/L, while the CrtZ variant from *Pantoea ananatis* achieved a zeaxanthin titer of 70.5 ± 10.8 µg/L. Additionally, we optimized carotenoid production by exploring enzyme fusion strategies for all three studied carotenoids and organelle compartmentalization specifically for enhancing astaxanthin synthesis. We further improved carotenoid production by integrating the optimal gene constructs into the yeast genome and deleting the *GAL80* gene, enabling the use of sucrose as a carbon source. The engineered strain Sp_Bc-Can001 *∆gal80* was evaluated in a 5 L bioreactor fermentation, achieving a notable canthaxanthin titer of 60.36 ± 1.51 mg/L using sucrose. This research conclusively establishes *S. cerevisiae* as a viable platform for efficient carotenoid biosynthesis and, for the first time in this yeast system, illustrates sucrose’s viability as a carbon source for canthaxanthin production. These findings pave the way for sustainable, cost-effective carotenoid production at an industrial scale.

## 1. Introduction

In recent years, the metabolic engineering of microorganisms for the sustainable and environmentally friendly production of natural compounds has attracted significant interest [1]. Carotenoids, such as canthaxanthin, zeaxanthin, and astaxanthin, are of particular value due to their antioxidant properties and widespread application in food, cosmetic, and pharmaceutical industries [2,3]. These carotenoids are traditionally obtained from plant or algal sources but can also be synthesized by genetically engineering yeast such as *Saccharomyces cerevisiae* [4].

Carotenoids, composed of C40 isoprene units, are commercially valuable due to their wide range of bioactive properties. For instance, beta-carotene is a precursor to vitamin A, essential for proper eye health, while lycopene and astaxanthin are powerful antioxidants associated with preventing cancers and atherosclerosis [5]. Despite their benefits, the production of carotenoids currently relies on chemical synthesis and extraction from plants, which pose risks to food management and biological safety. Microbial production represents a promising and sustainable alternative [6].

Astaxanthin, in particular, is known for its potent antioxidant activity and potential health benefits, including the prevention of atherosclerotic cardiovascular diseases, one of the most common causes of death globally [6]. It is predominantly produced by microalgae such as *Haematococcus pluvialis* and yeasts like *Phaffia rhodozyma*. Zeaxanthin, another xanthophyll commonly found in plants and some microorganisms, plays a crucial role in eye health by protecting against oxidative stress. Canthaxanthin, although less common, has applications in food and feed industries due to its colorant properties.

However, the efficient production of carotenoids in *S. cerevisiae* presents challenges due to the complexity of the biosynthetic pathways involved. It requires the optimization of precursor supply while minimizing byproducts and improving enzyme efficiency. Recent studies have demonstrated that enhancing enzyme expression or engineering metabolic pathways can significantly improve carotenoid yields [7]. The versatility and economic feasibility of *S. cerevisiae* make it a promising platform for commercial carotenoid production.

This work aims to fill the gaps in the field, particularly focusing on novel carbon sources and genetic engineering techniques. The use of sucrose, an abundant and cost-effective carbon source, marks a significant improvement over traditional substrates due to its availability and low cost. Further, this study explores innovative strategies like enzyme fusion and organelle compartmentalization to enhance carotenoid yields [8,9]. While enzyme fusion was applied across canthaxanthin, zeaxanthin, and astaxanthin, organelle compartmentalization specifically targeted astaxanthin, leading to compartment-specific synthesis.

By optimizing the carotenoid biosynthesis pathway and improving fermentation processes, this research seeks to establish a robust platform for industrial-scale carotenoid production. To the best of our knowledge, this research is the first to demonstrate that sucrose can be effectively used as a primary carbon source for canthaxanthin production in *S. cerevisiae*, rendering this approach both more economical and sustainable. Through the systematic screening of enzymatic variants and improving genetic engineering strategies, this study aims to establish *S. cerevisiae* as a versatile cell factory for high-value carotenoids, paving the way for future advances in sustainable bioprocessing.

## 2. Materials and Methods

### 2.1. Yeast Strain, Media, and Transformation

We generated engineered strains from the parent *S. cerevisiae* strains WWY005, Sp_Bc, FPPY005, and BeCaYeast [10,11]. The genotypes of the strains are provided in the Supplementary Materials. Codon-optimized coding sequences are provided in the Supplementary Materials. Plasmid construction was carried out using various techniques, including traditional restriction digestion followed by ligation, and yeast-based homologous recombination. The primers used for plasmid construction, strain construction, and strain verification are listed in Table S1. *Escherichia coli* DH5α served as the host for plasmid amplification. We utilized standard methodologies to prepare competent *E. coli* and *S. cerevisiae* cells, followed by their transformation as previously described [12,13]. For the construction of vectors, *E. coli* was cultured in Luria–Bertani (LB) broth (0.5% yeast extract, 1% peptone, and 0.5% sodium chloride). The validation of plasmid constructs was conducted using colony PCR and subsequent DNA sequencing. The yeast strains lacking plasmids were propagated in YPD medium (1% yeast extract, 2% peptone, and 2% glucose). For the selection and carotenoid production assays, yeast transformants were cultured on a minimal yeast medium consisting of Yeast Nitrogen Base (6.7 g/L), a carbon source of glucose (20 g/L), or a glucose mixture in a ratio of 0.2:1.8, with a total of 20 g/L carbon source for carotenoid biosynthesis, supplemented with a mix of essential nucleotide bases and amino acids, minus those specific to the selection markers used.

### 2.2. Plasmid Construction

Plasmid construction utilized the pRSII426-Gal1/10 backbone [11], which features a *GAL1/GAL10* bidirectional promoter, the *CPS1* terminator, and the *HIS5* terminator. To create *CrtW* expression plasmids, individual *CrtW* genes codon-optimized for *S. cerevisiae* expression were synthesized by GenScript and provided in pUC57 plasmids. These synthesized genes were designed with *BamH*I and *Sal*I restriction sites at the 5′ and 3′ ends, respectively. The CrtW gene fragments were amplified from pUC57-XxCrtW using primers CrtW-uni-BamHI-F and CrtW-uni-SalI-R. After purification, the fragments were inserted into the *BamH*I/*Sal*I site of the pRSII426-Gal1/10 backbone, resulting in individual *CrtW* expression plasmids like pRSII426-Gal1/10-BrevCrtW.

For *CrtZ* expression plasmids, individual *CrtZ* genes, also codon-optimized for *S. cerevisiae* expression, were synthesized by GenScript and supplied in pUC57 plasmids. These genes were designed to be flanked by *Spe*I and *EcoR*I restriction sites at the 5′ and 3′ ends, respectively. The *CrtZ* gene fragments were amplified from the pUC57-XxCrtZ plasmid using primers CrtZ-uni-SpeI-F and CrtZ-uni-EcoRI-R. The purified fragments were inserted into the *Spe*I/*EcoR*I site of pRSII426-Gal1/10, creating individual *CrtZ* expression plasmids such as pRSII426-Gal1/10-PaCrtZ.

Using this strategy, *CrtW* and *CrtZ* co-expression plasmids were also constructed. The *CrtW* gene was ligated into the *BamH*I/*Sal*I site and the *CrtZ* gene into the *Spe*I/*EcoR*I site of pRSII426-Gal1/10, resulting in co-expression plasmids like pRSII426-Gal1/10-BrevCrtW-PaCrtZ. For the construction of plasmids containing fused enzymes and those designed for organelle compartmentalization, detailed methodologies are provided in the Supplementary Materials.

### 2.3. Strain Construction

Strain BeCaYeast: The expression cassette for *SpCrtE*, *SpCrtYB*, and *SpCrtI* was amplified from pRSII416-loxp-Ura3-BCC39850-loxp-SpCrtE-SpCrtYB-SpCrtI using primers 1622_int_F_loxP and 1622_int_R_loxP. The purified DNA fragment was transformed into strain FPPY005 to create BeCaYeast. Colony PCR was used to verify the genomic integration using the following primers: 1622_ups_seq_F and 1622_dwst_seq_R. The Ura3 selectable marker was recycled using the loxP-Cre recombinase system [14].

Strain BeCaYeast-ARE: The expression cassette for *ScARE1* and *ScARE2* was amplified from pRSII416-loxp-Ura3-BCC39850-loxp-ScARE1-ScARE2 using primers 1414_int_F_loxP and 1414_int_R_loxP. The purified DNA fragment was transformed into strain BeCaYeast to create BeCaYeast-ARE. Colony PCR was used to verify the genomic integration using the following primers: 1414_upst_seq_F and 1414_dwst_seq_R. The Ura3 selectable marker was recycled using the loxP-Cre recombinase system.

Strain BeCaYeast-ARE-Can001: The expression cassette for *BradCrtW* and *SpCrtYB* as distinct enzymes was amplified from pRSII426-loxp-Ura3-BCC39850-loxp-BradCrtW-SpCrtYB using primers 511_int_F_loxP and 511_int_R_loxP. The purified DNA fragment was transformed into strain BeCaYeast-ARE. Colony PCR was used to verify the genomic integration using the following primers: 511_upst_seq_F and 511_dwst_seq_R. The Ura3 selectable marker was recycled using the loxP-Cre recombinase system. Strain Sp_Bc-Can001 was constructed using the same strategy, with the only difference being the background strain used (Sp_Bc instead of BeCaYeast-ARE).

Strain BeCaYeast-ARE-Zea001: The expression cassette for PaCrtZ-LF-SpCrtYB was amplified from pRSII426-loxp-Ura3-BCC39850-loxp-PaCrtZ-LF-SpCrtYB using primers 511_int_F_loxP and 511_int_R_loxP. The purified DNA fragment was transformed into strain BeCaYeast-ARE. Colony PCR was used to verify the genomic integration using the following primers: 511_upst_seq_F and 511_dwst_seq_R. The Ura3 selectable marker was recycled using the loxP-Cre recombinase system. Strain Sp_Bc-Zea001 was constructed using the same strategy, with the only difference being the background strain used (Sp_Bc instead of BeCaYeast-ARE).

Strain BeCaYeast-ARE-Asta001: The expression cassette for BradCrtW-GS-PaCrtZ was amplified from pRSII426-loxp-Ura3-BCC39850-loxp-BradCrtW-GS-PaCrtZ using primers 511_int_F_loxP and 511_int_R_loxP. The purified DNA fragment was transformed into strain BeCaYeast-ARE. Colony PCR was used to verify the genomic integration using the following primers: 511_upst_seq_F and 511_dwst_seq_R. The Ura3 selectable marker was recycled using the loxP-Cre recombinase system. Strain Sp_Bc-Asta001 was constructed using the same strategy, with the only difference being the background strain used (Sp_Bc instead of BeCaYeast-ARE).

Strain Sp_Bc-Asta002: The expression cassette for PaCrtZ-LF-SpCrtYB was amplified from pRSII416-loxp-Ura3-BCC39850-loxp-PaCrtZ-LF-SpCrtYB using primers 106_int_F_loxP and 106_int_R_loxP. The purified DNA fragment was transformed into strain Sp_Bc-Asta001. Colony PCR was used to verify the genomic integration using the following primers: 106_upst_seq_F and 106_dwst_seq_R. The Ura3 selectable marker was recycled using the loxP-Cre recombinase system.

*GAL80* Deletion Strains: The *GAL80* gene was deleted from strain Sp_Bc-Can001, Sp_Bc-Zea001 and Sp_Bc-Asta002 using the Cre/loxP recombination system, as described previously [10,14]. A donor sequence containing loxP-URA3-loxP, flanked by 50 bp homologous regions adjacent to the *GAL80* locus, was amplified using primers delGal80-F and delGal80-R with the pUG72 plasmid as a template. The purified DNA was transformed into Sp_Bc competent cells, which were selected on uracil-deficient SC medium. Transformation was confirmed by colony PCR using deltaGal80-seq-5end and Ura3_int_R primers. The *URA3* marker was removed from Sp_Bc *Δgal80* cells by transforming them with the pSH-Hyg plasmid encoding Cre recombinase. The pSH-Hyg plasmid was then eliminated by repeated culturing in YPD medium without hygromycin B. Successful transformants grew on SC medium containing 1 mg/mL 5-fluoroorotic acid but failed to grow on YPD medium with 200 µg/mL hygromycin B, confirming marker removal and plasmid loss.

### 2.4. Small Scale Fermentation

For fermentation in 50 mL conical tubes, strains were first precultured overnight in 5 mL of a minimal medium containing glucose as the sole carbon source. The preculture was used to inoculate a fresh 10 mL of minimal medium with 0.2% glucose and 1.8% galactose to achieve an initial OD_600_ of 0.05. After 72 h, 10 mL aliquots were collected and centrifuged at 12,000 rpm for 5 min. The resulting cell pellets were resuspended in 1.0 mL of 3N HCl, boiled for 5 min, cooled on ice for 5 min, and centrifuged at 12,000 rpm for 10 min. The pellets were washed once with 1 mL of deionized water before being resuspended in 1 mL of acetone with 1% butylated hydroxytoluene (BHT) and mixed with glass beads and vortexed vigorously. The samples were then centrifuged at 12,000 rpm for 10 min, and the cleared supernatant was filtered through a 0.2 μm Nylon membrane and transferred to amber HPLC vials.

In mixed sucrose–galactose experiments, the overnight culture was used to inoculate fresh 10 mL minimal medium with 2% sucrose and galactose at different ratios to achieve an initial OD_600_ of 0.05, following the same extraction procedure.

For fermentation in 250 mL shake-flasks, the overnight culture was used to inoculate 50 mL of YP medium (1% yeast extract and 2% peptone) containing either 2% sucrose (YPS) or a 2% mixed carbon source of sucrose and galactose at a 1:2 ratio (YPSG). Samples were collected at 24, 48, and 72 h for carotenoid quantification.

### 2.5. Quantitative Analysis of Carotenoids and Other Metabolites

Carotenoids were quantified using High-Performance Liquid Chromatography (HPLC), utilizing a Vanquish system equipped with a Hypersil GOLD^TM^ C18 column (4.6 mm × 150 mm), following a previously established methodology with minor adjustments [10]. The HPLC was operated with a gradient mobile phase consisting of acetonitrile at 90% (Solution B) and a mixture of methanol and isopropanol in a 3:2 ratio (Solution A). The analysis started with 100% Solution B, transitioning to 10% Solution B and 90% Solution A over 15 min. This composition was maintained for 15 min, followed by a ramp up to 100% Solution B within 5 min, where it was maintained for an additional 5 min. The flow rate was kept at 1.0 mL/min, with the column temperature maintained at 20 °C. Carotenoids—canthaxanthin, zeaxanthin, and astaxanthin—were detected using a diode array detector (DAD) set to a 450 nm wavelength. Carotenoid concentrations were established using calibration curves prepared for each standard obtained from Sigma Aldrich, according to the supplier’s instructions. Additional HPLC analysis for sugars and alcohols was performed using a Shodex SH1011 column (8.0 mm × 300 mm), with a mobile phase of 3 mM perchloric acid at a flow rate of 0.4 mL/min. Detection was achieved via the refractive index (RID), with the column maintained at 30 °C.

### 2.6. Bioprocess Development at the 5 L Scale

The optimization of fed-batch fermentation was conducted in a 5 L stirred-tank bioreactor (Biostat B, Sartorius, Germany), using a process derived and adapted from a previously established protocol [10]. The bioreactor fermentations were run in duplicate, and the data points represent the mean values from both runs, with error bars indicating the standard deviation. The initial batch comprised 2 L of a semi-defined medium, comprising 5 g/L ammonium sulfate, 3 g/L potassium dihydrogen phosphate, 0.5 g/L magnesium sulfate heptahydrate, 5 g/L yeast extract, 10 g/L peptone, and 1 mL/L trace metal solution (pH adjusted to 4.0). The trace metal solution was composed of the following: 15.0 g/L of EDTA (sodium form), 4.5 g/L of zinc sulfate heptahydrate, 1 g/L of manganese(II) chloride tetrahydrate, 0.3 g/L of cobalt(II) chloride hexahydrate, 0.3 g/L of copper(II) sulfate pentahydrate, 0.4 g/L of sodium molybdate dihydrate, 4.5 g/L of calcium chloride dihydrate, 3.0 g/L of iron(II) sulfate heptahydrate, 1.0 g/L of boric acid, and 0.10 g/L of potassium iodide. Inexpensive sucrose (Mitr Phol) served as a carbon source. Fed-batch stages commenced at the 10th hour, with additions of 5 g/L sucrose and 0.75 g/L yeast extract at 5 h intervals across 20 cycles. These periodic additions aimed to reach final concentrations of 120 g/L sucrose and an additional 15 g/L yeast extract, resulting in a total nitrogen source concentration of 30 g/L when combined with the initial 5 g/L yeast extract and 10 g/L peptone used in the batch phase. Preculture conditions were standardized for optimal inoculum density (OD_600_ 0.5). The fermentation pH was controlled at 5.0 using 2 N potassium hydroxide, with sugar levels carefully regulated to remain below threshold concentrations. Aerobic conditions were maintained with a 0.5 VVM aeration rate and 500 rpm agitation. All fermentations were kept at a constant 30 °C for optimal growth and production.

## 3. Results and Discussion

### 3.1. Optimizing Carotenoid Biosynthesis: Screening of CrtW and CrtZ Enzymatic Variants in S. cerevisiae

**Figure 1:**
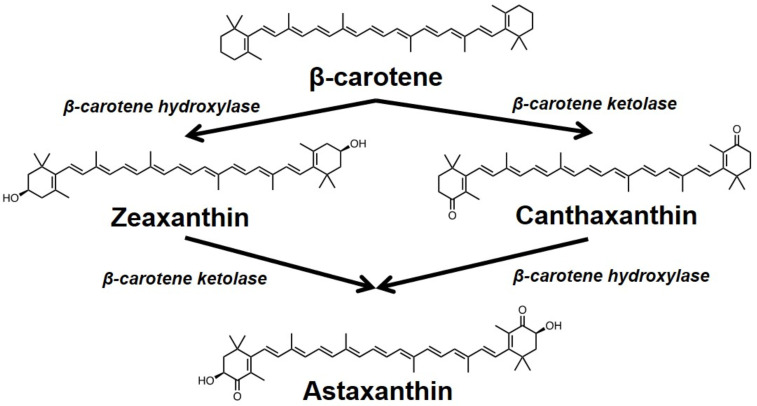
A schematic representation of engineered carotenoid biosynthesis in *S. cerevisiae*. The pathway initiates with the conversion of β-carotene into zeaxanthin through the enzymatic action of β-carotene hydroxylase (CrtZ). An alternative route involves β-carotene ketolase (CrtW) catalyzing the formation of canthaxanthin from β-carotene. The subsequent transformation of zeaxanthin to astaxanthin is facilitated by β-carotene ketolase, and conversely, astaxanthin can also be synthesized from canthaxanthin through the hydroxylation process mediated by β-carotene hydroxylase.

We initiated our investigation by identifying the most effective gene or gene combinations for introduction into *S. cerevisiae*. Our aim was to optimize the biosynthesis of high-value carotenoids, namely canthaxanthin, zeaxanthin, and astaxanthin, each with substantial therapeutic potential and increasing commercial demand (Figure 1).

Our experimental strategy utilized a specifically tailored parental yeast strain, Sp_Bc, which had been previously engineered to produce β-carotene [10]. This prolific strain served as an ideal host due to its enhanced mevalonate and carotenoid pathways, featuring the overexpression of genes *CrtE/YB/I* from the red yeast *Sporidiobolus pararoseus* TBRC-BCC 63403. With this robust platform in place, we employed β-carotene as the precursor for the targeted biosynthesis of the carotenoids in question.

The *CrtW* and *CrtZ* genes, codon-optimized for expression in *S. cerevisiae*, play critical roles in converting β-carotene to canthaxanthin and zeaxanthin, respectively [15,16]. Their codon optimization ensures robust expression in yeast while circumventing codon bias issues that can affect heterologous protein synthesis. A list of these genes and their sources is provided in Table 1. The astaxanthin production was pursued by co-expressing combinations of *CrtW* and *CrtZ* genes from the aforementioned list.

**Figure 2:**
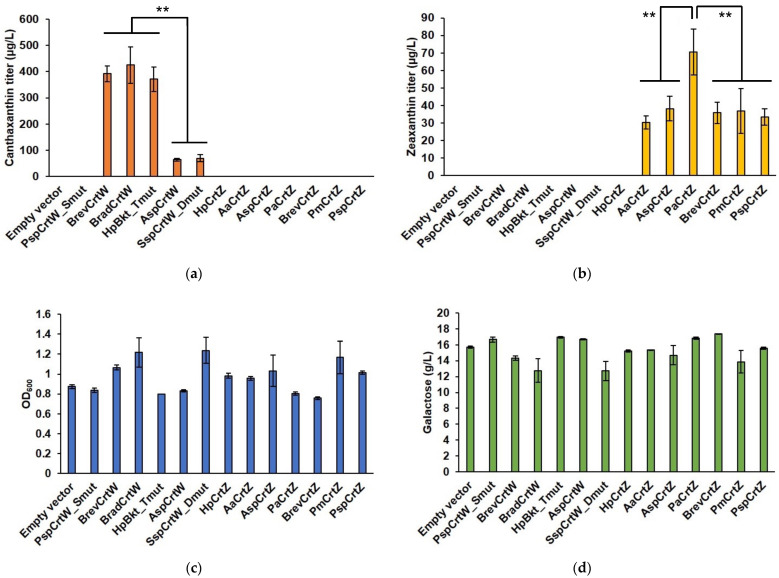
Carotenoid production and fermentation metrics in engineered yeast. (**a**) Canthaxanthin concentration achieved by genetically modified yeast strains; (**b**) zeaxanthin concentration measured in the same set of strains; (**c**) optical density (OD_600_) indicating cellular growth; (**d**) remaining concentration of galactose after a 72 h fermentation period. Engineered yeast strains were incubated in SCG-Ura medium with an initial mixture of 0.2% glucose and 1.8% galactose at 30 °C, with shaking at 250 rpm. Galactose levels were quantified using HPLC at the end of the fermentation process. Statistically significant differences are marked with ‘**’ (*p* < 0.05) using a two-tail, unpaired, heteroscedastic Student’s *t*-test.

Through the heterologous expression of CrtW and CrtZ variants sourced from a diverse pool of microorganisms, we were able to pinpoint enzymes with high conversion efficiency. The CrtW variants from *Brevundimonas vesicularis*, *Bradyrhizobium* sp., and a mutant form from *Haematococcus pluvialis* emerged as leading candidates for canthaxanthin production, delivering titers of 392.3 ± 30.4 µg/L, 425.1 ± 69.1 µg/L, and 370.9 ± 47.0 µg/L, respectively (Figure 2). Similarly, the CrtZ variant from *Pantoea ananatis* demonstrated superior efficiency in zeaxanthin synthesis, showing a 2.3-fold increase over the benchmark variant from *Agrobacterium aurantiacum* [17].

### 3.2. Enhanced Carotenoid Production through Enzyme Fusion

**Figure 3:**
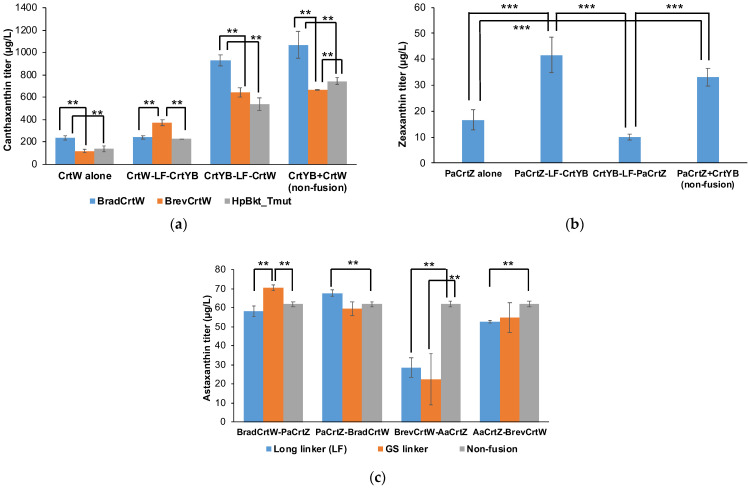
The effects of enzyme fusion on canthaxanthin (**a**), zeaxanthin (**b**), and astaxanthin (**c**) titers. Carotenoids were measured after a 72 h fermentation period. LF stands for the long GGGGSGGPGS linker, while GS stands for the short GS linker. The orientation of the fused enzymes is presented from the N-terminus to the C-terminus. Engineered yeast strains were incubated in SCG-Ura medium with an initial mixture of 0.2% glucose and 1.8% galactose at 30 °C, with shaking at 250 rpm. Experiments were conducted in triplicate, and values are presented as the mean ± standard deviation. Statistically significant differences are marked with ‘***’ (*p* < 0.01) of ‘**’ (*p* < 0.05) using a two-tail, unpaired, heteroscedastic Student’s *t*-test.

Building on our screening results of CrtW and CrtZ variants, we further explored the potential of enzyme fusion to boost carotenoid biosynthesis (Figure 3). This strategy was aimed at enhancing the proximity of sequential enzymatic reactions, thereby potentially increasing the efficiency of substrate channeling between enzymatic steps.

For canthaxanthin, we designed fusion constructs combining SpCrtYB, a bifunctional enzyme crucial for lycopene cyclization and β-carotene synthesis, with the following best-performing ketolase variants: BradCrtW, BrevCrtW, and the HpBkt mutant. To explore the effects of enzyme orientation on biosynthesis, SpCrtYB was positioned at both the N-terminus and C-terminus of these constructs. A flexible, glycine-rich linker (GGGGSGGPGS; LF linker) was employed to connect the enzymes, chosen for its minimal interference with protein folding and function [18]. For zeaxanthin production, we adopted a similar strategy, creating fusions of SpCrtYB with PaCrtZ. These constructs were also tested in both orientations to determine the optimal arrangement for zeaxanthin synthesis.

All fused enzymes were expressed in the BeCaYeast-ARE yeast strain. BeCaYeast-ARE has the same genetic modifications as strain Sp_Bc, with two key differences. First, *ScARE1* and *ScARE2* are overexpressed in BeCaYeast-ARE. These genes, involved in sterol ester synthesis, have been shown to improve carotenoid production in yeast [19]. Second, the background strain for BeCaYeast-ARE is TBRC-BCC39850, known for its tolerance to pretreatment inhibitors [20]. In contrast, Sp_Bc uses CEN.PK2-1C, a common laboratory strain. The screening of these enzyme fusions was conducted using plasmid-based expression systems, which allowed for the rapid assembly and modification of genetic constructs. This method facilitated a swift comparative analysis of different enzyme configurations and their impact on carotenoid production. As controls in our experiments, we also overexpressed the individual enzymes—CrtW and CrtZ—as distinct entities within the same BeCaYeast-ARE background. This approach enabled us to directly assess the added value of the fusion strategy compared to traditional overexpression methods.

Our results showed that the non-fused combination of BradCrtW and SpCrtYB achieved the highest canthaxanthin titer of 1068 ± 118 µg/L, while the SpCrtYB-LF-BradCrtW fusion construct reached 927 ± 51 µg/L (Figure 3a). Other fusion constructs, SpCrtYB-LF-BrevCrtW and SpCrtYB-LF-HpBkt, produced titers of 642 ± 40 µg/L and 537 ± 57 µg/L, respectively. For zeaxanthin production, the non-fused PaCrtZ and SpCrtYB combination reached 33 ± 3 µg/L, whereas SpCrtYB-LF-PaCrtZ achieved 10 ± 1 µg/L (Figure 3b). Interestingly, PaCrtZ-LF-SpCrtYB reached a higher titer of 42 ± 7 µg/L.

Introducing an additional copy of SpCrtYB, particularly with BradCrtW for canthaxanthin, led to higher production levels than fusion constructs (Figure 3a). This outcome indicates that key enzyme availability in the biosynthetic pathway is more critical than their proximity in a fused protein. The limited success of enzyme fusions could be attributed to structural changes that impact enzyme functionality, the increased metabolic burden of synthesizing large fusion proteins, or suboptimal linker configurations [21]. These findings emphasize the complexities of metabolic engineering via protein fusion and highlight the importance of revisiting expression strategies. Enhancing native enzyme efficiency or using co-expression systems without fusion may optimize valuable compound production in microbial hosts.

### 3.3. Construction of Astaxanthin-Producing Yeast Strain

After investigating enzyme fusion strategies for canthaxanthin and zeaxanthin production, we shifted focus to engineering yeast for astaxanthin production by co-expressing CrtW and CrtZ. Our investigations expanded to the co-expression of the efficient beta-carotene ketolase and hydroxylase enzymes, BradCrtW and PaCrtZ, respectively, identified from preliminary screening experiments. As benchmarks, we also evaluated the BrevCrtW and AaCrtZ combination, previously noted for effective astaxanthin production in *S. cerevisiae* [17]. We utilized a single plasmid with bidirectional galactose-inducible *GAL1/10* promoters to co-express these enzymes, both as standalone and as fused proteins. This strategy, aimed at increasing metabolic pathway efficiency through substrate channeling or compartmentalizing metabolic pathways, has shown potential in enhancing product titers by minimizing issues like toxic intermediates and slow reaction rates.

Given the mixed success of enzyme fusion for canthaxanthin and zeaxanthin production, we also explored whether this strategy could improve astaxanthin titers (Figure 3c). We generated plasmids for co-expressing the selected CrtW and CrtZ genes with both short GS linkers and long, flexible glycine-rich GGGGSGGPGS linkers. Following the findings by Hu and coworkers [22], which highlighted the importance of fusion order on enzymatic function, we constructed eight different fusion plasmid configurations. These were transformed into the yeast strain BeCaYeast-ARE, alongside controls expressing the *CrtW* and *CrtZ* genes separately.

Astaxanthin production was quantitatively assessed (Figure 3c). The BradCrtW-GS-PaCrtZ construct produced a titer of 70.39 ± 1.49 µg/L, outperforming other configurations. The long-linker (LF) construct of BradCrtW-LF-PaCrtZ achieved 58.26 ± 2.81 µg/L, while the non-fused version reached 61.75 ± 1.11 µg/L. Other PaCrtZ-BradCrtW fusions ranged between 59.52 µg/L and 67.51 µg/L. BrevCrtW-AaCrtZ fusions ranged from 22.40 µg/L to 28.42 µg/L, and AaCrtZ-BrevCrtW produced between 52.64 µg/L and 54.64 µg/L. These results indicate that while enzyme fusion marginally outperformed separate expression, it did not consistently deliver significant gains. The slight variations in titers among constructs show a minimal advantage of enzyme fusion.

### 3.4. Compartmentalizing Astaxanthin Biosynthesis: Targeting Organelles

Given the mixed success of enzyme fusion to enhance astaxanthin production, we explored enzyme compartmentalization as an alternative strategy [23,24]. Drawing from the success of compartmentalization in oleaginous yeast (*Yarrowia lipolytica*) [25], we targeted specific organelles (peroxisomes, endoplasmic reticulum, and lipid bodies) in *S. cerevisiae* to enhance enzyme activity and substrate accessibility. To specifically direct the fused enzymes to these organelles, we utilized distinct targeting signals: The Ser-Lys-Leu (SKL) signal peptide was added to the C-terminus for peroxisome localization, exploiting their oxidative environment [26]. The Trp-Glu-His-Asp-Glu-Leu (WEHDEL) sequence directed enzymes to the endoplasmic reticulum, leveraging its extensive membrane network for potential efficiency in substrate channeling [27]. For lipid bodies, the *Zea mays* L. oleosin (ZmOle) protein was fused to the C-terminus, aiming to utilize their hydrophobic nature as a sink for astaxanthin accumulation [28].

**Figure 4:**
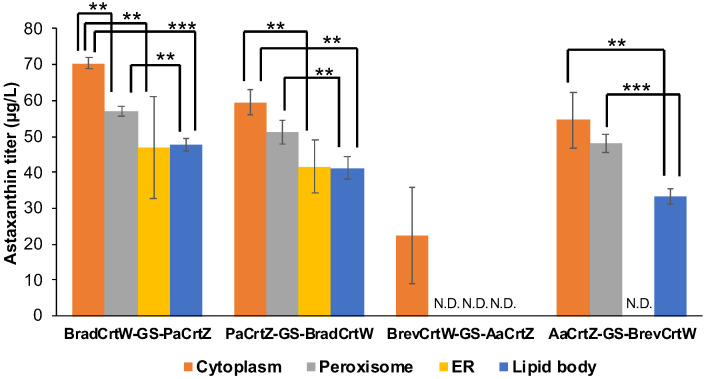
The effects of enzyme compartmentalization on astaxanthin titer. GS stands for the short GS linker. The orientation of the fused enzymes is presented from the N-terminus to the C-terminus. Carotenoids were measured after a 72 h fermentation period. Engineered yeast strains were incubated in SCG-Ura medium with 0.2% glucose and 1.8% galactose at 30 °C, with shaking at 250 rpm. Experiments were conducted in triplicate, and values are presented as the mean ± standard deviation. N.D. means not detected. Statistically significant differences are marked with ‘***’ (*p* < 0.01) of ‘**’ (*p* < 0.05) using a two-tail, unpaired, heteroscedastic Student’s *t*-test.

The results showed varied responses to compartmentalization (Figure 4). Enzymes localized in the cytoplasm consistently produced the highest astaxanthin titers compared to other organelles. For example, the BradCrtW-GS-PaCrtZ fused enzymes achieved a titer of 70.39 ± 1.49 µg/L in the cytoplasm, compared to 57.00 ± 1.52 µg/L in peroxisomes, 46.99 ± 14.09 µg/L in the endoplasmic reticulum, and 47.62 ± 1.83 µg/L in lipid bodies. PaCrtZ-GS-BradCrtW also performed best in the cytoplasm, reaching 59.52 ± 3.56 µg/L, compared to 51.21 ± 3.41 µg/L in peroxisomes, 41.63 ± 7.43 µg/L in the endoplasmic reticulum, and 41.27 ± 3.14 µg/L in lipid bodies.

In contrast, the BrevCrtW-GS-AaCrtZ fused enzymes demonstrated poor performance outside the cytoplasm, yielding 22.40 ± 13.58 µg/L in the cytoplasm, while no astaxanthin was detected when these enzymes were targeted to peroxisomes or the endoplasmic reticulum. Similarly, AaCrtZ-GS-BrevCrtW achieved a titer of 54.64 ± 7.79 µg/L in the cytoplasm and 48.08 ± 2.68 µg/L in peroxisomes, while no astaxanthin was detected when these enzymes were targeted to the endoplasmic reticulum and only 33.28 ± 2.16 µg/L in lipid bodies.

These results suggest that organelle targeting is not universally beneficial and is influenced by the specific metabolic and organelle dynamics of the yeast strain. The compartmentalization strategy that works in *Y. lipolytica* does not necessarily translate well to *S. cerevisiae*. The lack of improvement in astaxanthin titers could be due to differences in lipid accumulation, organelle interactions, and metabolic flux between oleaginous and non-oleaginous yeasts.

### 3.5. Integration of Carotenoid Biosynthetic Genes into Yeast Genome

In our pursuit to optimize carotenoid production in yeast, we identified the optimal CrtW and CrtZ enzymes for their superior activity within the yeast context. The influence of protein fusion on production titer was systematically evaluated, leading us to select the most effective gene constructs for integration into the yeast genome. This transition from plasmid-based expression to genomic integration offers numerous advantages, including enhanced genomic stability and the elimination of the need for continuous selective pressure, thus simplifying cultivation and potentially increasing the viability of industrial applications [29].

For canthaxanthin production, the optimal setup involved expressing SpCrtYB and BradCrtW as separate proteins. In the case of zeaxanthin, the results between a fused enzyme configuration with PaCrtZ at the N-terminus and SpCrtYB at the C-terminus and the non-fused enzymes were not statistically significant (*p*-value = 0.07). However, we chose to integrate the fused enzyme construct due to its potential advantages in substrate channeling, which could enhance overall pathway efficiency. For astaxanthin production, the preferred arrangement was a cytosol-targeted fusion of BradCrtW and PaCrtZ, with BradCrtW at the N-terminus and PaCrtZ at the C-terminus, using the short GS linker. These constructs were integrated into the *ARS511* locus, known for its favorable expression levels and integration efficiency [30].

We evaluated the performance of these engineered constructs in two different yeast backgrounds: BeCaYeast-ARE and Sp_Bc. The latter demonstrated significantly better performance, leading to its selection for further experiments. In quantitative terms, the canthaxanthin-producing strain Sp_Bc-Can001 produced canthaxanthin at a titer of 14.12 ± 1.66 mg/L, significantly outperforming the BeCaYeast-ARE-derived strain which produced only 1.08 ± 0.05 mg/L. Similarly, for zeaxanthin, Sp_Bc-Zea001 produced 1.29 ± 0.05 mg/L, vastly exceeding the output from BeCaYeast-ARE-Zea001, which managed just 0.03 ± 0.00 mg/L. For astaxanthin production, the BeCaYeast-ARE-Asta001 strain did not produce detectable levels, whereas the Sp_Bc-Asta001 strain managed a modest production of 44 ± 2 µg/L. To enhance astaxanthin yield, we integrated an additional copy of the SpCrtYB and PaCrtZ fusion into the Sp_Bc-Asta001 strain at the *ARS106* locus, creating the Sp_Bc-Asta002 strain. This modification significantly improved astaxanthin production to 479.01 ± 24.36 µg/L.

When comparing our astaxanthin results to previous studies, it is evident that our achieved astaxanthin titers are lower. For instance, the engineered strain SyBE_Sc2110M3 produced 218 mg/L [17], Yast-TS14 achieved 235 mg/L [31], and AX15 reached 404.78 mg/L [32] in fed-batch fermentation. These studies employed extensive genetic engineering strategies to reach higher titers.

The strain SyBE_Sc2110M3 was created by overexpressing *CRTE, CRTYB*, and *CRTI* from *Xanthophyllomyces dendrorhous* in multiple copies [17]. Additionally, the overexpression of *tHMG1* and a fusion gene *BTS1-ERG20*, along with the plasmid-based overexpression of *CRTW* from *Brevundimonas vesicularis* and *CRTZ* from *Agrobacterium aurantiacum*, significantly contributed to the enhanced carotenoid production. Furthermore, ARTP (Atmospheric and Room-Temperature Plasma) mutagenesis was employed to improve strain performance. The strain Yast-TS14 was developed by overexpressing *CRTE03M* (one copy), *CRTYB* (three copies), and *CRTI* (two copies) from *X. dendrorhous* [31]. This strain also included the overexpression of *OCRTZM1* (three copies) and *OBKTM29* (one copy) from *Haematococcus pluvialis*, *tHMG1*, and *GAL4M9*. Key gene deletions such as *GAL80, GAL1, GAL4, GAL7, GAL10, YPL062W, LPP1*, and *DPP1* were implemented to optimize the metabolic pathways. Directed evolution and temperature-responsive regulation further enhanced the strain’s efficiency. The strain AX15 was engineered by overexpressing *CRTE* (one copy) from *Taxus media*, *CARRA* (two copies), and *CRTI* (two copies) from *Blakeslea trispora* [32]. Additionally, it included the overexpression of *tHMG1*, *CRTW* (one copy) from *B. vesicularis*, and *CRTZ* (one copy) from *A. aurantiacum*. Deletions of *YPL062W*, *GAL1*, *GAL7*, and *GAL10* were performed to enhance pathway efficiency. Both ARTP and H_2_O_2_ mutagenesis were utilized to achieve higher titers.

These genetic engineering strategies, combined with directed evolution and mutagenesis techniques, enabled these strains to achieve several hundred milligrams of astaxanthin per liter in fed-batch fermentation. In future studies, applying similar strategies to our engineered strains could significantly enhance their productivity, potentially reaching titers comparable to those reported in the literature.

### 3.6. GAL80 Deletion and Its Impact on Carotenoid Production

The next phase of our study involves developing a bioprocess for canthaxanthin production using sucrose, a cheap and readily available carbon source, instead of relying on the more expensive galactose to induce the *GAL* promoters. While galactose is effective for inducing the *GAL* promoters, it is costly when scaling up production [33]. *GAL80* encodes a transcriptional repressor regulating the cell’s transcriptional response to galactose [34]. In *S. cerevisiae*, Gal80p interacts with the *GAL* promoters, and, when galactose is present, Gal80p is sequestered away from Gal4p, lifting the repression and activating the promoter [35]. However, glucose (and sucrose by extension) represses this system through Mig1p and Mig2p [36,37].

**Figure 5:**
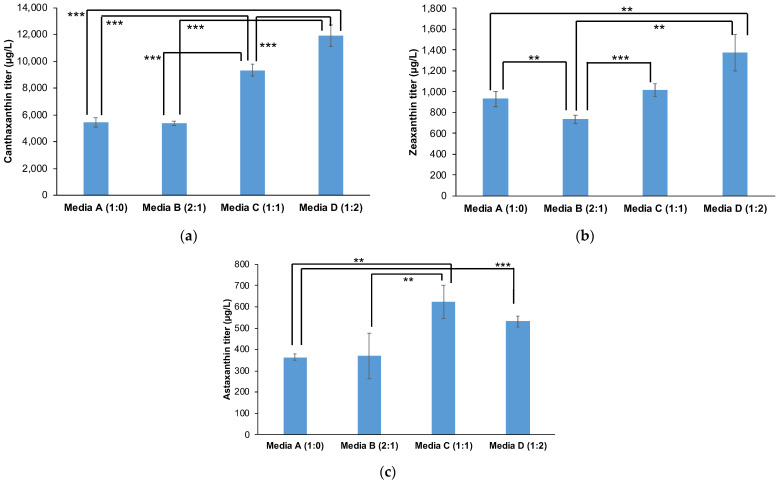
Carotenoid production in 50 mL conical tubes across varying sucrose-to-galactose ratios. The fermentation of strains Sp_Bc-Can001 *∆gal80*, Sp_Bc-Zea001 *∆gal80*, and Sp_Bc-Asta002 *∆gal80* in 50 mL conical tubes using media with different sucrose-to-galactose ratios. Four sucrose-to-galactose ratios were tested: Media A, 100% sucrose; Media B, 2:1 ratio of sucrose to galactose; Media C, 1:1 ratio; and Media D, 1:2 ratio, maintaining a total concentration of 2%. (**a**) Canthaxanthin production in strain Sp_Bc-Can001 *∆gal80*. (**b**) Zeaxanthin production in strain Sp_Bc-Zea001 *∆gal80*. (**c**) Astaxanthin production in strain Sp_Bc-Asta002 *∆gal80*. The experiments were conducted in triplicate, with results presented as the mean ± standard deviation. Statistically significant differences are marked with ‘***’ (*p* < 0.01) of ‘**’ (*p* < 0.05) using a two-tail, unpaired, heteroscedastic Student’s *t*-test.

By deleting *GAL80*, we intend to uncouple carotenoid production from galactose dependence, enabling the use of sucrose as a cost-effective alternative. Consequently, we deleted *GAL80* from the three selected strains, Sp_Bc-Can001, Sp_Bc-Zea001, and Sp_Bc-Asta002, resulting in Sp_Bc-Can001 *∆gal80*, Sp_Bc-Zea001 *∆gal80*, and Sp_Bc-Asta002 *∆gal80*. We then evaluated their carotenoid production in sucrose-containing media using 50 mL conical tubes (Figure 5). Four different sucrose-to-galactose ratios were tested: (A) sucrose as the sole carbon source, (B) sucrose–galactose at a 2:1 ratio, (C) sucrose–galactose at a 1:1 ratio, and (D) sucrose–galactose at a 1:2 ratio. The total concentration of these combined carbon sources was maintained at 2% for all ratios.

A clear production trend was observed: as the proportion of galactose increased, production levels for all three carotenoids rose accordingly. The highest yields were consistently obtained at ratio D (sucrose–galactose at a 1:2 ratio), suggesting that galactose remains crucial for maximizing production even with the *GAL80* deletion. This influence may be due to galactose’s involvement in other metabolic pathways or regulatory mechanisms not entirely bypassed by the *GAL80* deletion. Despite this, the production levels achieved with sucrose-dominant ratios demonstrate the potential for further optimization to develop a cost-effective bioprocess using sucrose as a primary carbon source.

### 3.7. Bioprocess Development for Canthaxanthin Production Using Sucrose

**Figure 6:**
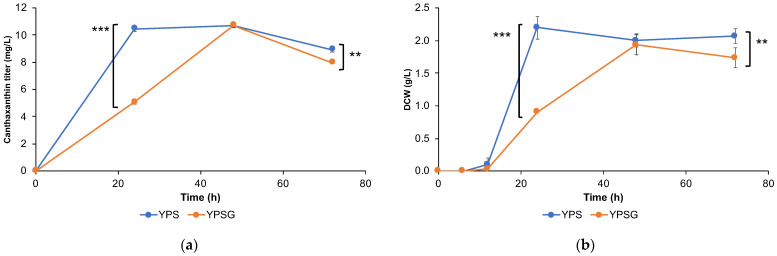
The shake-flask fermentation of strain Sp_Bc Can001 *∆gal80* in yeast medium containing either 2% sucrose as the sole carbon source (YPS) or a 2% mixed carbon source (sucrose and galactose at a 1:2 ratio) (YPSG). (**a**) Canthaxanthin production (mg/L). (**b**) Biomass accumulation as dry cell weight (DCW, in g/L). The experiments were conducted in triplicate, and values are presented as the mean ± standard deviation. Statistically significant differences are marked with ‘***’ (*p* < 0.01) of ‘**’ (*p* < 0.05) using a two-tail, unpaired, heteroscedastic Student’s *t*-test.

To confirm the scalability of our engineered strains, we developed a bioprocess for canthaxanthin production using Sp_Bc-Can001 *∆gal80*. We scaled up to 50 mL in 250 mL shake-flasks using two different carbon source conditions: (1) 2% sucrose as the sole carbon source and (2) a 2% mixed carbon source of sucrose and galactose at a 1:2 ratio (Figure 6). The peak canthaxanthin production of around 10.71 mg/L was observed at 48 h under both conditions. Production declined by 72 h, likely due to the instability of canthaxanthin caused by photo-oxidation. Based on these findings, we chose sucrose as the sole carbon source for subsequent 5 L bioreactor experiments because it is significantly cheaper (Mitr Phol: 0.67 USD/kg) compared to galactose (Difco: 276 USD/kg).

**Figure 7:**
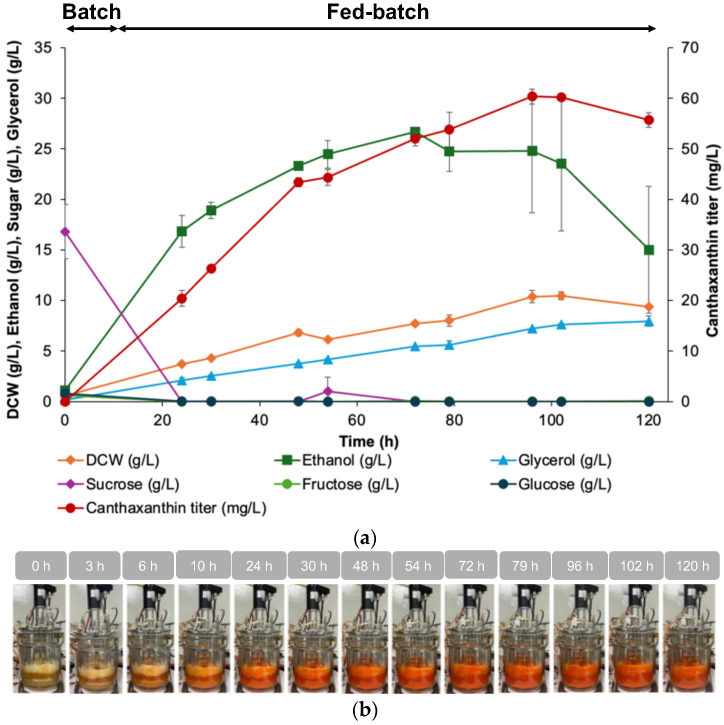
The fed-batch fermentation of strain Sp_Bc Can001 *∆gal80* in a 5 L fermenter using sucrose as the sole carbon source. (**a**) The fermentation profile including canthaxanthin titer, biomass as dry cell weight (DCW), ethanol production, glycerol production, and residual sugars (sucrose, fructose, and glucose) over time. Data points represent the averages and standard deviations from duplicate runs. The fermentation began with a 2 L batch phase using a basal salt medium supplemented with 20 g/L sucrose. The fed-batch stage started at the 10th hour, with periodic sucrose additions at 5 h intervals across 20 cycles, cumulatively reaching 120 g/L of sucrose. (**b**) The visual progression of the bioreactor culture over time, with an intensifying red/orange hue reflecting the increasing canthaxanthin concentration, providing a visual confirmation of successful carotenoid biosynthesis.

In 5 L fed-batch bioreactor experiments, the maximum canthaxanthin titer of 60.36 ± 1.51 mg/L was achieved at 96 h, before declining to 55.73 ± 1.42 mg/L at the end of the fermentation (120 h) (Figure 7). Ethanol production peaked at 26.71 ± 0.00 g/L at 72 h, indicating significant fermentation byproducts. Glycerol also accumulated, reaching 7.94 ± 0.52 g/L at the end of the process. Residual sugar analysis showed that sucrose was fully consumed after the 24 h mark, while minor amounts of fructose and glucose remained. The significant ethanol and glycerol production indicate a redox imbalance within the cells, possibly due to the heterologous expression of the carotenoid pathway. This imbalance could shift the cells to produce more fermentation byproducts. Metabolic engineering strategies to alleviate this could include reducing ethanol formation by modifying fermentative pathways or introducing redox-balancing enzymes to maintain cellular redox homeostasis.

In previous studies, significant advancements in the production of canthaxanthin using engineered strains of *S. cerevisiae* have been reported. For example, Chen et al. (2022) achieved canthaxanthin production titers of up to 1.44 g/L by introducing the β-carotene ketolase variant OBKTM29 into a β-carotene producer, with further enhancements through the subcellular re-localization and overexpression of pleiotropic drug resistance (PDR) regulators to improve stress tolerance [38].

Despite these impressive results, our study employs sucrose, a cheap and renewable carbon source, for canthaxanthin production, which significantly reduces the cost of the fermentation process. This approach aligns with the sustainable and economical production goals, though the achieved titers in our study are lower compared to the aforementioned studies. In Chen et al. (2022), the engineering strategy included the introduction of multiple copies of carotenoid biosynthetic genes and the re-localization of enzymes to specific cellular compartments to optimize metabolic flux. Our approach similarly focuses on enzyme targeting and integration into the yeast genome to stabilize the expression and improve production efficiency. However, unlike the extensive genetic modifications and iterative optimization applied in other studies, our current work provides a foundational approach that can be further enhanced through similar iterative processes including directed evolution and mutagenesis strategies.

Moreover, Chen et al. (2022) also explored the overexpression of PDR regulators *Pdr1* and *Pdr3* to improve the stress tolerance of the yeast strain, leading to enhanced canthaxanthin production. In their follow-up study, they investigated the roles of PDR regulators and discovered that the overexpression of Pdr3p boosted carotenoid biosynthesis by activating *GAL* promoters [39]. This mechanism, revealed through comparative transcriptomics, reverse metabolic engineering, and electrophoretic mobility shift assay (EMSA), suggests that Pdr3p can enhance the transcriptional levels of *GAL* promoter-driven genes by binding to their upstream activation sequences (UASs).

Future work could integrate these advanced engineering strategies into our existing framework to achieve higher canthaxanthin titers. By combining our sustainable approach using sucrose with further strain optimization, including the overexpression of PDR regulators and employing directed evolution or ARTP mutagenesis, we can build upon our current achievements and explore avenues for further improvement in the production of high-value carotenoids.

## 4. Conclusions

In this study, we engineered *S. cerevisiae* to produce high-value carotenoids such as canthaxanthin, zeaxanthin, and astaxanthin. Through a systematic screening of CrtW and CrtZ enzymatic variants, we identified optimal candidates and evaluated enzyme fusion strategies to enhance biosynthesis. While enzyme fusion did not always yield expected improvements, the overexpression of key enzymes like SpCrtYB and BradCrtW significantly boosted production levels. By integrating optimal gene constructs into the yeast genome, we achieved stable carotenoid production. Our deletion of *GAL80* uncoupled carotenoid synthesis from the costly inducer galactose, enabling the use of sucrose as a carbon source. Scaling up to a 5 L bioreactor confirmed that sucrose, a cheap and renewable substrate, supported a canthaxanthin titer of 60.36 ± 1.51 mg/L, marking the first demonstration of sucrose’s viability as a carbon source for canthaxanthin production in the *S. cerevisiae* system. These findings pave the way for a further exploration of agricultural waste products like molasses or raw sugar as potential carbon sources, creating opportunities for circular economy principles while reducing production costs and environmental impact.

## Figures and Tables

**Table 1:** CrtW and CrtZ candidates from bacteria and microalgae.

Name	Source	Mutant/Wild-Type	GenBank Accession No.
PspCrtW_Smut	*Paracoccus* sp. N81106	L175W	AB206672
BrevCrtW	*Brevundimonas vesicularis*	Wild-type	DQ309446.1
BradCrtW	*Bradyrhizobium* sp. ORS278	Wild-type	AF218415.1
HpBkt_Tmut	*Haematococcus pluvialis*	H165R/V264D/F298Y	KP866870.1
AspCrtW	*Alcaligenes* sp. PC-1	Wild-type	D58422.1
SspCrtW_Dmut	*Sphingomonas* sp. DC18	R203W/F213L	DQ400932.1
HpCrtZ	*Haematococcus pluvialis*	Wild-type	KP866868
AaCrtZ	*Agrobacterium aurantiacum*	Wild-type	GM621472.1
AspCrtZ	*Alcaligenes* sp. PC-1	Wild-type	D58422.1
PaCrtZ	*Pantoea ananatis*	Wild-type	D90087
BrevCrtZ	*Brevundimonas* sp. SD212	Wild-type	AB181388.1
PmCrtZ	*Paracoccus marcussii*	Wild-type	MT175370.1
PspCrtZ	*Paracoccus* sp. N81106	Wild-type	AB206672

## Data Availability

The original contributions presented in the study are included in the article/Supplemenatry Materials, further inquiries can be directed to the corresponding author.

## Supplementary Materials

The following supporting information can be downloaded at https://www.mdpi.com/article/10.3390/jof10060433/s1, Detailed description of plasmid and strain construction; genotypes of strains used in this study; codon-optimized coding sequences used in this study; Table S1: Primers used in this study.
