# Supplementary material for: Metabolic Engineering of Saccharomyces cerevisiae for Production of Canthaxanthin, Zeaxanthin, and Astaxanthin
Source: J Fungi (Basel). 2024 Jun 18;10(6):433. doi: 10.3390/jof10060433 (PMC11205050; doi:10.3390/jof10060433)
Supplement: Supplementary file 1 [file jof-10-00433-s001.zip › jof-3035696-supplementary.pdf]

Supporting Information

Table S1. Primers used in this study

Primer Name	Primer Sequence (5’ to 3’)
delUra3-39850-hyg-F	TGGTGAAGGATAAGTTTTGACCATCAAAGAAGGTTATAACTTCGTATAGCATACATTATACGAAGTTATCCAGC GACATGGAGGCCC
delUra3-39850-hyg-R	TTTCTTTCCAATTTTTTTTTTTTCGTCATTATAAAATAACTTCGTATAATGTATGCTATAC GAAGTTATCAGTTTTCGACACTGGATGGC
LoxP-Ura-XhoI-F	ATCTCGAGATAACTTCGTATAGCATACATTATACGAAGTTATAATGTGGCTGTGGTTTCA
LoxP-Ura-SalI-R	TATGTCGACATAACTTCGTATAATGTATGCTATACGAAGTTATAATCATTACGACCGAGA
CrtW-Uni-BamHI-F	CTAGATATCGGATCCAAAACAATG
CrtW-Uni-SalI-R	CAGGCCTCTGCAGTCGAC
CrtZ-Uni-SpeI-F	GGATCCGATACTAGTAAAACAATG
CrtZ-Uni-EcoRI-R	ACGACGGCCAGTGAATTC
Crt-Univ-BamHI-F	ACAATAGGATCCAAAACAATG
Crt-Univ-SalI-R	GATCCGATTATGTCGAC
BradW_LgFs_PaZ_R1	AGAGCCGGGTCCGCCAGAGCCTCCGCCGCCATCTCTTCTTTCCAATGCTCT
BradW_LgFs_PaZ_F2	GGCGGCGGAGGCTCTGGCGGACCCGGCTCTATGTTGTGGATCTGGAACG
BradW_LgFs_PaZ_SalI_ R2	ATATGTCGACTTATTTACCTGATGCTGGTTC
BrevW_LgFs_AaZ_R1	AGAGCCGGGTCCGCCAGAGCCTCCGCCGCCAGAAAACAAAGACCACCATG
BrevW_LgFs_AaZ_F2	GGCGGCGGAGGCTCTGGCGGACCCGGCTCTATGGCAAATTTCTTGATCGTT
BrevW_LgFs_AaZ_SalI_ R2	ATATGTCGACTTATGTTCTTTCTTGTGCTTC
AaZ_LgFs_BrevW_BamH I_F1	ATATGGATCCAAAACAATGGCAAATTTCTTG
AaZ_LgFs_BrevW_R1	AGAGCCGGGTCCGCCAGAGCCTCCGCCGCCTGTTCTTTCTTGTGCTTCAG
AaZ_LgFs_BrevW_F2	GGCGGCGGAGGCTCTGGCGGACCCGGCTCTATGTCAGCTGTTACTCCAAT
PaZ_LgFs_BradW_BamH I_F1	ATATGGATCCAAAACAATGTTGTGGATCTGG
PaZ_LgFs_BradW_R1	AGAGCCGGGTCCGCCAGAGCCTCCGCCGCCTTTACCTGATGCTGGTTCAT
PaZ_LgFs_BradW_F2	GGCGGCGGAGGCTCTGGCGGACCCGGCTCTATGCATGCAGCTACAGCA
BradW_GS_PaZ_R1	GATCCACAACATagaaccATCTCTTCTTTCCAATGCTCT
BradW_GS_PaZ_F2	GAAAGAAGAGATggttctATGTTGTGGATCTGGAACG

BrevW_GS_AaZ_R1	GAAATTTGCCATagaaccAGAAAACAAAGACCACCATG
BrevW_GS_AaZ_F2	TCTTTGTTTTCTggttctATGGCAAATTTCTTGATCGTT
AaZ_GS_BrevW_R1	AACAGCTGACATagaaccTGTTCTTTCTTGTGCTTCAG
AaZ_GS_BrevW_F2	CAAGAAAGAACAggttctATGTCAGCTGTTACTCCAAT
PaZ_GS_BradW_R1	AGCTGCATGCATagaaccTTTACCTGATGCTGGTTCAT
PaZ_GS_BradW_F2	GCATCAGGTAAAggttctATGCATGCAGCTACAGCA
BrevW_LgFs_SpYB_F2	GGCGGCGGAGGCTCTGGCGGACCCGGCTCTatgggtttcgattactggtt
BrevW_LgFs_SpYB_R2	ATATGTCGACttataaagctctccatgctgt
PaZ-WEHDEL-SalI-R	TATAGTCGACTTATAGTTCATCATGTTCCCATTTACCTGATGCTGGTTCATC
AaZ-WEHDEL-SalI-R	TATAGTCGACTTATAGTTCATCATGTTCCCATGTTCTTTCTTGTGCTTCAG
BrevW-WEHDEL-SalI-R	TATAGTCGACTCATAGTTCATCATGTTCCCAAGAAAACAAAGACCACCATG
BradW-WEHDEL-SalI-R	TATAGTCGACTCATAGTTCATCATGTTCCCAATCTCTTCTTTCCAATGCTC
PaZ-ZmOle-R	AGAGCCGGGTCCGCCAGAGCCTCCGCCGCCTTTACCTGATGCTGGTTCATC
AaZ-ZmOle-R	AGAGCCGGGTCCGCCAGAGCCTCCGCCGCCTGTTCTTTCTTGTGCTTCAG
BrevW-ZmOle-R	AGAGCCGGGTCCGCCAGAGCCTCCGCCGCCAGAAAACAAAGACCACCATG
BradW-ZmOle-R	AGAGCCGGGTCCGCCAGAGCCTCCGCCGCCATCTCTTCTTTCCAATGCTC
LF-ZmOle-F	GGCGGCGGAGGCTCTGG
LF-ZmOle-SalI-R	TATAGTCGACTCAGGATGAAGCTCTACCACC
1622_int_F_loxP	AATATTGTAGTGCAGAAGGTAACAGCAAAAACAAATAGTTCACCCCCCCCTCGAGATAAC
1622_int_R_loxP	GGAATTAGTTTATTAGAGGAAGTGCCAGGCGACATAAAGTTCCGCTCTAGAACTAGTATC
1622_ups_seq_F	TCCTAATTGTGTTTTATCCG
1622_dwst_seq_R	ATTCCTGCTTCGATTGAG
TEF1-ARE2-F	TAGAAAGAAAGCATAGCAATCTAATCTAAGTTTAAAACAATGGACAAGAAGAAGGATCTA
TEF1-ARE2-R	GCCTGGTAAAGTTGTGTGCTAGTGTCTCCCGTCTTCTGTTTAGAATGTCAAGTACAACGT
TEF1-TDH3-F1	TCGTTCTCCAGTAGATCCTTCTTCTTGTCCATTGTTTTAAACTTAGATTAGATTGCTATG
TEF1-TDH3-R1	CGTATTCTTTGAAATGGCAGTATTGATAATGATAAACTCATAGCTTCAAAATGTTTCTAC
TEF1-TDH3-F2	GGAAGAGTAAAAAAGGAGTAGAAACATTTTGAAGCTATGAGTTTATCATTATCAATACTG
TEF1-TDH3-R2	AACTCTTCGTCTTGCAACAAATCCTTAGTCTCCGTCATTGTTTTGTTTGTTTATGTGTGT
TDH3-ARE1-F	AAGAACTTAGTTTCGAATAAACACACATAAACAAACAAAACAATGACGGAGACTAAGGAT
TDH3-ARE1-R	AAAATTAAAAAAAAAAAATCTTTGACTATTCAATCATTGCGCTCATAAGGTCAGGTACAA
1622_dwst_seq_R	ATTCCTGCTTCGATTGAG
1622 dwst R_new	TGTTTATGGGTGTCATCACCC
1414_int_F_loxP	GTAATCTGGCATTTCATACTATCATTGCTCAAATTATCCGCCGCCCCCCCTCGAGATAAC
1414_int_R_loxP	TCTTTTGTTCTTTTTCTTTTTTTGGAGATGCGTCTTAAAAACCGCTCTAGAACTAGTATC
1414_upst_seq_F	TCCAAAAAAGTGGTTCATTCG
1414_dwst_seq_R	CCTTACTCCCAAGCAATAAACG
511_int_F_loxP	GAGAGAAGACTAAAAATATCTGCTGCAAGAACAAAAATGGGGCGAATTGGGTACCATAAC
511_int_R_loxP	TTTCATAGCATTCAAAAACCGTTGCTGTGTAAAAAAAGTCgctcAAATTCATCCTCTATC

511_upst_seq_F	TATCTGAGCATACCCGCC
511_dwst_seq_R	GTGTTGAGCGTGTTAGCTG
106_int_F_loxP	AAAAAAAAAATAGCCGCCATGACCTCGGATCGTCGGTTGTGGCGAATTGGGTACCATAAC
106_int_R_loxP	TTTCTTCCTTGTTATTTTTCTTAGTTCTGAAGTTTGACCAgctcAAATTCATCCTCTATC
106_ups_seq_F	AGGTGAATTATTTTTCCCCCC
106_dwst_seq_R	GCGAAAATCTTTCTGCCGG
delGal80_F	AATCTCGATAGTTGGTTTCCCGTTCTTTCCACTCCCGTCATGCATAGGCCACTAGTGGAT
delGal80_R	GTTCGCTGCACTGGGGGCCAAGCACAGGGCAAGATGCTTTTACAGCTGAAGCTTCGTACG
delGal80_seq_5end	AAGAAAATCACACGAGCG
Ura3_int_R	AATTGGTCTTCTTTTCATCC

Genotypes of strains used in this study

WWY005: CEN.PK2-1C*, 416d::HIS3_*T_{HMG1}*-tHMGR-*P_{GAL1}*-*P_{GAL10}*-ERG12-*T_{ERG12}*, 308a::LEU2_*T_{HMG1}*-tHMGR-*P_{GAL1}*-*P_{GAL10}*-ERG8-*T_{ERG8}*, 720a::TRP1_*T_{HMG1}*-tHMGR-*P_{GAL1}*-*P_{GAL10}*-ERG19-*T_{ERG19}*, SAP155c::loxP_*T_{ERG13}*-ERG13-*P_{GAL1}*-*P_{GAL10}*-IDI1-*T_{IDI1}*, YPRCd15c::loxP_*T_{ERG10}*-ERG10-*P_{GAL1}*-*P_{GAL10}*-ERG20-*T_{ERG20}.

Sp_Bc: WWY005 *1021b::*T_{PRM9}*-SpCrtE(opt)-*P_{GAL1}*-*P_{GAL10}*-SpCrtYB(opt)-*T_{GAL10}*-*P_{GAL7}*-SpCrtI(opt)-*T_{CPS1}.

FPPY005: BCC39850, *his3Δ; leu2Δ; ura3Δ; YPRCΔ15c::loxP_*T_{ERG10}*-ERG10-*P_{GAL1}*-*P_{GAL10}*-ERG20-*T_{ERG20}*; ARS308a:: loxP_*T_{HMG1}*-tHMGR-*P_{GAL1}*-*P_{GAL10}*-ERG8-*T_{ERG8}*; ARS1021::loxP_*T_{ERG13}*-ERG13-*P_{GAL1}*-*P_{GAL10}*-IDI1-*T_{IDI1}*; ARS720a::loxP_*T_{HMG1}*-tHMGR-*P_{GAL1}*-*P_{GAL10}*-ERG19-*T_{ERG19}*; ARS1309::loxP_*T_{HMG1}*-tHMGR-*P_{GAL1}*-*P_{GAL10}*-ERG12-*T_{ERG12}

BeCaYeast: FPPY005 *1622::*T_{PRM9}*-SpCrtE(opt)-*P_{GAL1}*-*P_{GAL10}*-SpCrtYB(opt)-*T_{GAL10}*-*P_{GAL7}*-SpCrtI(opt)-*T_{CPS1}.

Plasmid pRSII416-loxp-Ura3-BCC39850-loxp-SpCrtE-SpCrtYB-SpCrtI: The Ura3 selectable marker was amplified from the genomic DNA of TBRC-BCC39850 using primers LoxP-Ura-XhoI-F and LoxP-Ura-SalI-R. Both of these primers contain the LoxP sequence. The purified fragment was ligated to the *Xho*I/*Sal*I sites of pRSII416-*crt* (Watcharawipas *et al.*, 2021) to create pRSII416-loxp-Ura3-BCC39850-loxp-SpCrtE-SpCrtYB-SpCrtI.

Plasmid pRSII426-PaCrtZ-pGAL1/10-BrevCrtW: The β-carotene ketolase gene from *Brevundimonas vesicularis* (*BrevCrtW*) codon optimized for yeast expression was amplified from the pUC57-BrevCrtW using primers CrtW-uni-BamHI-F and CrtW-uni-SalI-R. The purified fragment was ligated to the *BamH*I/*Sal*I site of pRSII426-Gal1/10-PaCrtZ to create pRSII426-PaCrtZ-pGAL1/10-BrevCrtW.

Plasmid pRSII426-PaCrtZ-pGAL1/10-BradCrtW: The β-carotene ketolase gene from *Bradyrhizobium* sp. ORS278 (*BradCrtW*) codon optimized for yeast expression was amplified from the pUC57-BradCrtW using primers CrtW-uni-BamHI-F and CrtW-uni-SalI-R. The purified fragment was ligated to the *BamH*I/*Sal*I site of pRSII426-Gal1/10-PaCrtZ to create pRSII426-PaCrtZ-pGAL1/10-BradCrtW.

Plasmid pRSII426-SpCrtYB-pGAL1/10-BradCrtW: The β-carotene ketolase gene from *Bradyrhizobium* sp. ORS278 (*BradCrtW*) codon optimized for yeast expression was amplified from the pUC57-BradCrtW using primers CrtW-uni-BamHI-F and CrtW-uni-SalI-R. The purified fragment was ligated to the *BamH*I/*Sal*I site of pRSII426-Gal1/10 to create pRSII426-pGAL1/10-BradCrtW. *SpCrtYB* codon optimized for yeast expression was amplified from the pUC57-SpCrtYB using primers SpCrtYBCodOp-SpeI-F and SpCrtYBCodOp-EcoRI-R. The purified fragment was ligated to the *EcoR*I/*Spe*I sites of pRSII426-pGAL1/10-BradCrtW to create pRSII426-SpCrtYB-pGAL1/10-BradCrtW.

Plasmid pRSII426-loxp-Ura3-BCC39850-loxp-SpCrtYB-pGAL1/10-BradCrtW: The Ura3 selectable marker was amplified from the genomic DNA of TBRC-BCC39850 using primers LoxP-Ura-XhoI-F and LoxP-Ura-SalI-R. Both of these primers contain the LoxP sequence. The purified fragment was ligated to the *Xho*I/*Sal*I sites of pRSII426-SpCrtYB-pGAL1/10-BradCrtW to create pRSII426-loxp-Ura3-BCC39850-loxp-SpCrtYB-pGAL1/10-BradCrtW.

Plasmid pRSII426-PaCrtZ-pGAL1/10-HpBkt-Tmut: The β-carotene ketolase gene from *Haematococcus pluvialis* with the triple mutations H165R/V264D/F298Y (*$HpBkt_{H165R/V264D/F298Y}$*) codon optimized for yeast expression was amplified from the pUC57-HpBkt-Tmut using primers CrtW-uni-BamHI-F and CrtW-uni-SalI-R. The purified fragment was ligated to the *BamH*I/*Sal*I site of pRSII426-Gal1/10-PaCrtZ to create pRSII416-PaCrtZ-pGAL1/10-HpBkt-Tmut.

Plasmid pRSII426-Gal1/10-BradCrtW-GGGGSGGPGS-PaCrtZ: The β-carotene ketolase gene from *Bradyrhizobium* sp. ORS278 (*BradCrtW*) codon optimized for yeast expression was amplified from pUC57-BradCrtW using primers CrtW-uni-BamHI-F and BradW_LgFs_PaZ_R1. The β-carotene hydroxylase gene from *Pantoea ananatis* (*PaCrtZ*) codon optimized for yeast expression was amplified from pUC57-PaCrtZ using primers BradW_LgFs_PaZ_F2 and BradW_LgFs_PaZ_SalI_R2. These two DNA fragments were assembled together by overlap-extension PCR (OE-PCR). The resulting cassette was ligated to the *BamH*I/*Sal*I sites of pRSII426-Gal1/10 to create pRSII426-Gal1/10-BradCrtW-GGGGSGGPGS-PaCrtZ.

Plasmid pRSII426-Gal1/10-BradCrtW-GS-PaCrtZ: The β-carotene ketolase gene from *Bradyrhizobium* sp. ORS278 (*BradCrtW*) codon optimized for yeast expression was amplified from pUC57-BradCrtW using primers CrtW-uni-BamHI-F

and BradW_GS_PaZ_R1. The β-carotene hydroxylase gene from *Pantoea ananatis* (*PaCrtZ*) codon optimized for yeast expression was amplified from pUC57-PaCrtZ using primers BradW_GS_PaZ_F2 and BradW_GS_PaZ_SalI_R2. These two DNA fragments were assembled together by overlap-extension PCR (OE-PCR). The resulting cassette was ligated to the *BamH*I/*Sal*I sites of pRSII426-Gal1/10 to create pRSII426-Gal1/10-BradCrtW-GS-PaCrtZ.

Plasmid pRSII426-loxp-Ura3-BCC39850-loxp-Gal1/10-BradCrtW-GS-PaCrtZ: The Ura3 selectable marker was amplified from the genomic DNA of TBRC-BCC39850 using primers LoxP-Ura-XhoI-F and LoxP-Ura-SalI-R. Both of these primers contain the LoxP sequence. The purified fragment was ligated to the *Xho*I/*Sal*I sites of pRSII426-Gal1/10-BradCrtW-GS-PaCrtZ to create pRSII426-loxp-Ura3-BCC39850-loxp-Gal1/10-BradCrtW-GS-PaCrtZ.

Plasmid pRSII426-Gal1/10-PaCrtZ-GGGGSGGPGS-BradCrtW: The β-carotene hydroxylase gene from *Pantoea ananatis* (*PaCrtZ*) codon optimized for yeast expression was amplified from pUC57-PaCrtZ using primers CrtZ-uni-SpeI-F and PaZ_LgFs_BradW_R1. The β-carotene ketolase gene from *Bradyrhizobium* sp. ORS278 (*BradCrtW*) codon optimized for yeast expression was amplified from pUC57-BradCrtW using primers PaZ_LgFs_BradW_F2 and Crt-Univ-SalI-R. These two DNA fragments were assembled together by overlap-extension PCR (OE-PCR). The resulting cassette was ligated to the *BamH*I/*Sal*I sites of pRSII426-Gal1/10 to create pRSII426-Gal1/10-PaCrtZ-GGGGSGGPGS-BradCrtW.

Plasmid pRSII426-Gal1/10-PaCrtZ-GGGGSGGPGS-ScCrtYB: The β-carotene hydroxylase gene from *Pantoea ananatis* (*PaCrtZ*) codon optimized for yeast expression was amplified from pUC57-PaCrtZ using primers CrtZ-uni-SpeI-F and PaZ_LgFs_BradW_R1. *SpCrtYB* codon optimized for yeast expression was amplified from the pUC57-SpCrtYB using primers BrevW_LgFs_SpYB_F2 and BrevW_LgFs_SpYB_R2. These two DNA fragments were assembled together by overlap-extension PCR (OE-PCR). The resulting cassette was ligated to the *BamH*I/*Sal*I sites of pRSII426-Gal1/10 to create pRSII426-Gal1/10-PaCrtZ-GGGGSGGPGS-ScCrtYB.

Plasmid pRSII426-loxp-Ura3-BCC39850-loxp-Gal1/10-PaCrtZ-GGGGSGGPGS-ScCrtYB: The Ura3 selectable marker was amplified from the genomic DNA of TBRC-BCC39850 using primers LoxP-Ura-XhoI-F and LoxP-Ura-SalI-R. Both of these primers contain the LoxP sequence. The purified fragment was ligated to the *Xho*I/*Sal*I sites of pRSII426-Gal1/10-PaCrtZ-GGGGSGGPGS-ScCrtYB to create pRSII426-loxp-Ura3-BCC39850-loxp-Gal1/10-PaCrtZ-GGGGSGGPGS-ScCrtYB.

Plasmid pRSII426-Gal1/10-PaCrtZ-GS-BradCrtW: The β-carotene hydroxylase gene from *Pantoea ananatis* (*PaCrtZ*) codon optimized for yeast expression was amplified from pUC57-PaCrtZ using primers CrtZ-uni-SpeI-F and

PaZ_GS_BradW_R1. The β-carotene ketolase gene from *Bradyrhizobium* sp. ORS278 (*BradCrtW*) codon optimized for yeast expression was amplified from pUC57-BradCrtW using primers PaZ_GS_BradW_F2 and Crt-Univ-SalI-R. These two DNA fragments were assembled together by overlap-extension PCR (OE-PCR). The resulting cassette was ligated to the *BamH*I/*Sal*I sites of pRSII426-Gal1/10 to create pRSII426-Gal1/10-PaCrtZ-GS-BradCrtW.

Plasmid pRSII426-Gal1/10-BrevCrtW-GGGGSGGPGS-AaCrtZ: The β-carotene ketolase gene from *Brevundimonas vesicularis* (*BrevCrtW*) codon optimized for yeast expression was amplified from pUC57-BrevCrtW using primers CrtW-uni-BamHI-F and BrevW_LgFs_PaZ_R1. The β-carotene hydroxylase gene from *Agrobacterium aurantiacum* (*AaCrtZ*) codon optimized for yeast expression was amplified from pUC57-AaCrtZ using primers BrevW_LgFs_AaZ_F2 and BrevW_LgFs_AaZ_SalI_R2. These two DNA fragments were assembled together by overlap-extension PCR (OE-PCR). The resulting cassette was ligated to the *BamH*I/*Sal*I sites of pRSII426-Gal1/10 to create pRSII426-Gal1/10-BrevCrtW-GGGGSGGPGS-AaCrtZ.

Plasmid pRSII426-Gal1/10-BrevCrtW-GS-AaCrtZ: The β-carotene ketolase gene from *Brevundimonas vesicularis* (*BrevCrtW*) codon optimized for yeast expression was amplified from pUC57-BrevCrtW using primers CrtW-uni-BamHI-F and BrevW_GS_PaZ_R1. The β-carotene hydroxylase gene from *Agrobacterium aurantiacum* (*AaCrtZ*) codon optimized for yeast expression was amplified from pUC57-AaCrtZ using primers BrevW_GS_AaZ_F2 and BrevW_GS_AaZ_SalI_R2. These two DNA fragments were assembled together by overlap-extension PCR (OE-PCR). The resulting cassette was ligated to the *BamH*I/*Sal*I sites of pRSII426-Gal1/10 to create pRSII426-Gal1/10-BrevCrtW-GS-AaCrtZ.

Plasmid pRSII426-Gal1/10-AaCrtZ-GGGGSGGPGS-BrevCrtW: The β-carotene hydroxylase gene from *Agrobacterium aurantiacum* (*AaCrtZ*) codon optimized for yeast expression was amplified from pUC57-AaCrtZ using primers CrtZ-uni-SpeI-F and AaZ_LgFs_BrevW_R1. The β-carotene ketolase gene from *Brevundimonas vesicularis* (*BrevCrtW*) codon optimized for yeast expression was amplified from pUC57-BrevCrtW using primers AaZ_LgFs_BrevW_F2 and Crt-Univ-SalI-R. These two DNA fragments were assembled together by overlap-extension PCR (OE-PCR). The resulting cassette was ligated to the *BamH*I/*Sal*I sites of pRSII426-Gal1/10 to create pRSII426-Gal1/10-AaCrtZ-GGGGSGGPGS-BrevCrtW.

Plasmid pRSII426-Gal1/10-AaCrtZ-GS-BrevCrtW: The β-carotene hydroxylase gene from *Agrobacterium aurantiacum* (*AaCrtZ*) codon optimized for yeast expression was amplified from pUC57-AaCrtZ using primers CrtZ-uni-SpeI-F and AaZ_GS_BrevW_R1. The β-carotene ketolase gene from *Brevundimonas vesicularis* (*BrevCrtW*) codon optimized for yeast expression was amplified from pUC57-BrevCrtW using primers AaZ_GS_BrevW_F2 and Crt-Univ-SalI-R. These

two DNA fragments were assembled together by overlap-extension PCR (OE-PCR). The resulting cassette was ligated to the *BamH*I/*Sal*I sites of pRSII426-Gal1/10 to create pRSII426-Gal1/10-AaCrtZ-GS-BrevCrtW.

Plasmid pRSII426-Gal1/10-BradCrtW-GS-PaCrtZ-GGGGSGGPGS-ZmOle: The oleosin gene from *Zea mays* (*ZmOle*) was codon-optimized for *S. cerevisiae* expression. The gene was synthesized by GenScript and was provided on a pUC57 plasmid. The *ZmOle* gene fragment was amplified from the pUC57-ZmOle using primers LF-ZmOle-F and LF-ZmOle-SalI-R. The BradCrtW-GS-PaCrtZ fragment was amplified from pRSII426-Gal1/10-BradCrtW-GS-PaCrtZ using primers CrtW-uni-BamHI-F and PaZ-ZmOle-R. These two DNA fragments were assembled together by overlap-extension PCR (OE-PCR). The resulting cassette was ligated to the *BamH*I/*Sal*I sites of pRSII426-Gal1/10 to create pRSII426-Gal1/10-BradCrtW-GS-PaCrtZ-GGGGSGGPGS-ZmOle.

Plasmid pRSII426-Gal1/10-PaCrtZ-GS-BradCrtW-GGGGSGGPGS-ZmOle: The *ZmOle* gene fragment was amplified from the pUC57-ZmOle using primers LF-ZmOle-F and LF-ZmOle-SalI-R. The PaCrtZ-GS-BradCrtW fragment was amplified from pRSII426-Gal1/10-PaCrtZ-GS-BradCrtW using primers CrtW-uni-BamHI-F and BradW-ZmOle-R. These two DNA fragments were assembled together by overlap-extension PCR (OE-PCR). The resulting cassette was ligated to the *BamH*I/*Sal*I sites of pRSII426-Gal1/10 to create pRSII426-Gal1/10-PaCrtZ-GS-BradCrtW-GGGGSGGPGS-ZmOle.

Plasmid pRSII426-Gal1/10-BrevCrtW-GS-AaCrtZ-GGGGSGGPGS-ZmOle: The *ZmOle* gene fragment was amplified from the pUC57-ZmOle using primers LF-ZmOle-F and LF-ZmOle-SalI-R. The BrevCrtW-GS-AaCrtZ fragment was amplified from pRSII426-Gal1/10-BrevCrtW-GS-AaCrtZ using primers CrtW-uni-BamHI-F and AaZ-ZmOle-R. These two DNA fragments were assembled together by overlap-extension PCR (OE-PCR). The resulting cassette was ligated to the *BamH*I/*Sal*I sites of pRSII426-Gal1/10 to create pRSII426-Gal1/10-BrevCrtW-GS-AaCrtZ-GGGGSGGPGS-ZmOle.

Plasmid pRSII426-Gal1/10-AaCrtZ-GS-BrevCrtW-GGGGSGGPGS-ZmOle: The *ZmOle* gene fragment was amplified from the pUC57-ZmOle using primers LF-ZmOle-F and LF-ZmOle-SalI-R. The AaCrtZ-GS-BrevCrtW fragment was amplified from pRSII426-Gal1/10-AaCrtZ-GS-BrevCrtW using primers CrtW-uni-BamHI-F and BrevW-ZmOle-R. These two DNA fragments were assembled together by overlap-extension PCR (OE-PCR). The resulting cassette was ligated to the *BamH*I/*Sal*I sites of pRSII426-Gal1/10 to create pRSII426-Gal1/10-AaCrtZ-GS-BrevCrtW-GGGGSGGPGS-ZmOle

Plasmid pRSII416-ScARE2-TEF1/TDH3-ScARE1: The ScARE1, ScARE2, TEF1p and TDH3p fragments were amplified from BCC39850's genomic DNA using primers TDH3-ARE1-F and TDH3-ARE1-R, TEF1-ARE2-F and TEF1-ARE2-R, TEF1-TDH3-F1 and TEF1-TDH3-R1, TEF1-TDH3-F2 and TEF1-TDH3-R2, respectively. The four DNA fragments were

purified and assembled together with HpaI/AgeI digested pRSII416-*crt* (Watcharawipas *et al.*, 2021) via homologous recombination to form pRSII416-ScARE2-TEF1/TDH3-ScARE1.

Codon-optimized coding sequences used in this study

PspCrtW_Smut
ATGTCAGCACATGCTTTACCAAAAGCTGATTTGACAGCTACTTCATTGATTGTTTCTGGTGGTATTATTGCTGCATGGT
TAGCTTTGCATGTTCATGCTTTGTGGTTTTTGGATGCAGCTGCACATCCAATTTTGGCAATTGCTAATTTCTTGGGTTT
AACTTGGTTGTCTGTTGGTTTGTTTATTATTGCTCATGATGCTATGCATGGTTCTGTTGTTCCAGGTAGACCAAGAGCT
AATGCTGCAATGGGTCAATTGGTTTTATGGTTGTACGCAGGTTTTTCTTGGAGAAAAATGATTGTTAAACATATGGCTC
ATCATAGACATGCTGGTACTGATGATGATCCAGATTTTGATCATGGTGGTCCAGTTAGATGGTACGCTAGATTCATTG
GTACTTACTTTGGTTGGAGAGAAGGTTTGTTATTGCCAGTTATTGTTACTGTTTACGCTTTGATTTTAGGTGACAGATG
GATGTACGTTGTTTTCTGGCCATTGCCATCTATTTTGGCTTCTATTCAATGGTTTGTTTTTGGTACTTGGTTGCCACATA
GACCAGGTCATGATGCTTTTCCAGATAGACATAATGCAAGATCATCAAGAATTTCTGATCCAGTTTCTTTGTTAACTTG
TTTTCATTTTGGTGGTTACCATCATGAACATCATTTGCATCCAACTGTTCCATGGTGGAGATTGCCATCTACAAGAACT
AAAGGTGACACTGCT

BrevCrtW
ATGTCAGCTGTTACTCCAATGTCAAGAGTTGTTCCAAATCAAGCATTGATTGGTTTAACTTTGGCTGGTTTAATTGCTG
CTGCATGGTTAACTTTGCATATTTACGGTGTTTATTTTCATAGATGGACTATTTGGTCTGTTTTGACTGTTCCATTGATT
GTTGCTGGTCAAACTTGGTTGTCAGTTGGTTTGTTTATTGTTGCACATGATGCTATGCATGGTTCTTTAGCTCCAGCAA
GACCAAGATTGAATACTGCTATTGGTTCTTTGGCTTTGGCATTATACGCTGGTTTTAGATTCACACCATTGAAAACTGC
ACATCATGCTCATCATGCTGCACCAGGTACTGCTGATGATCCAGATTTTCATGCAGATGCTCCAAGAGCATTTTTACC
ATGGTTTTACGGTTTCTTTAGAACTTACTTTGGTTGGAGAGAATTGGCTGTTTTAACTGTTTTGGTTGCAGTTGCTGTTT
TGATTTTAGGTGCTAGAATGCCAAATTTGTTAGTTTTCTGGGCAGCTCCAGCATTATTGTCTGCTTTACAATTGTTTACA
TTTGGTACTTGGTTGCCACATAGACATACAGATGATGCTTTTCCAGATAATCATAATGCTAGAACTTCTCCATTTGGTC
CAGTTTTGTCTTTATTGACTTGTTTTCATTTTGGTAGACATCATGAACATCATTTGACTCCATGGAAACCATGGTGGTCT
TTGTTTTCT

BradCrtW
ATGCATGCAGCTACAGCAAAAGCTACTGAATTTGGTGCTTCAAGAAGAGATGATGCTAGACAAAGAAGAGTTGGTTTA
ACTTTGGCAGCTGTTATTATTGCTGCATGGTTGGTTTTACATGTTGGTTTGATGTTTTTCTGGCCATTGACTTTACATTC
TTTGTTACCAGCTTTGCCATTAGTTGTTTTGCAAACATGGTTGTACGTTGGTTTGTTTATTATTGCTCATGATTGTATGC

ATGGTTCTTTGGTTCCATTCAAACCACAAGTTAATAGAAGAATTGGTCAATTATGTTTGTTTTTATACGCTGGTTTTTCA
TTTGATGCTTTGAATGTTGAACATCATAAACATCATAGACATCCAGGTACAGCTGAAGATCCAGATTTTGATGAAGTTC
CACCACATGGTTTTTGGCATTGGTTTGCTTCATTTTTCTTGCATTATTTTGGTTGGAAACAAGTTGCTATTATTGCAGCT
GTTTCTTTGGTTTATCAATTGGTTTTTGCTGTTCCATTGCAAAATATTTTATTGTTTTGGGCTTTACCAGGTTTGTTATCT
GCATTACAATTGTTTACATTTGGTACTTACTTGCCACATAAACCAGCTACTCAACCATTTGCTGATAGACATAATGCTA
GAACTTCTGAATTTCCAGCTTGGTTATCTTTGTTAACTTGTTTTCATTTTGGTTTTCATCATGAACATCATTTGCATCCA
GATGCTCCATGGTGGAGATTGCCAGAAATTAAAAGAAGAGCATTGGAAAGAAGAGAT

HpBkt_Tmut
ATGCATGTTGCATCAGCTTTGATGGTTGAACAAAAAGGTTCTGAAGCAGCTGCTTCATCTCCAGATGTTTTGAGAGCA
TGGGCTACACAATACCATATGCCATCTGAATCTTCAGATGCAGCTAGACCAGCATTGAAACATGCTTACAAACCACCA
GCTTCAGATGCTAAAGGTATTACTATGGCTTTGACTATTATTGGTACTTGGACAGCTGTTTTCTTGCATGCTATTTTTCA
AATTAGATTGCCAACTTCAATGGATCAATTGCATTGGTTGCCAGTTTCAGAAGCTACAGCACAATTATTGGGTGGTTCA
TCTTCATTGTTGCATATTGCTGCAGTTTTTATTGTTTTGGAATTTTTATATACTGGTTTGTTTATTACTACACATGATGCA
ATGCATGGTACTATTGCTTTAAGACATAGACAATTGAATGATTTGTTAGGTAATATTTGTATTTCTTTGTACGCTTGGTT
TGATTACTCTATGTTGAGAAGAAAACATTGGGAACATCATAATCATACAGGTGAAGTTGGTAAAGATCCAGATTTTCAT
AAAGGTAATCCAGGTTTGGTTCCATGGTTTGCTTCTTTTATGTCTTCATACATGTCTTTGTGGCAATTTGCAAGATTGG
CTTGGTGGGCTGTTGTTATGCAAATGTTGGGTGCTCCAATGGCAAATTTGTTAGTTTTTATGGCTGCAGCTCCAATTTT
GTCTGCTTTTAGATTGTTTTATTTTGGTACTTACTTGCCACATAAACCAGAACCAGGTCCAGCTGCAGGTTCTCAAGAT
ATGGCTTGGTTTAGAGCTAAAACTTCAGAAGCATCTGATGTTATGTCATTTTTGACATGTTACCATTTTGATTTGCATTG
GGAACATCATAGATGGCCATACGCACCATGGTGGCAATTGCCACATTGTAGAAGATTATCAGGTAGAGGTTTAGTTCC
AGCTTTGGCT

AspCrtW
ATGTCAGGTAGAAAACCAGGTACTACAGGTGACACTATTGTTAATTTGGGTTTAACTGCTGCAATTTTGTTATGTTGGT
TGGTTTTGCATGCTTTTACTTTGTGGTTGTTAGATGCAGCTGCTCATCCATTGTTAGCTGTTTTGTGTTTAGCTGGTTT
GACTTGGTTGTCTGTTGGTTTGTTTATTATTGCTCATGATGCAATGCATGGTTCTGTTGTTCCAGGTAGACCAAGAGCA
AATGCTGCAATTGGTCAATTGGCTTTATGGTTGTACGCTGGTTTTTCATGGCCAAAATTGATTGCTAAACATATGACTC
ATCATAGACATGCTGGTACTGATAATGATCCAGATTTTGGTCATGGTGGTCCAGTTAGATGGTACGGTTCTTTTGTTTC
AACTTATTTTGGTTGGAGAGAAGGTTTGTTGTTACCAGTTATTGTTACAACTTACGCTTTGATTTTAGGTGACAGATGG
ATGTACGTTATTTTCTGGCCAGTTCCAGCAGTTTTGGCTTCTATTCAAATTTTTGTTTTTGGTACTTGGTTGCCACATAG
ACCAGGTCATGATGATTTTCCAGATAGACATAATGCTAGATCAACAGGTATTGGTGACCCATTGTCTTTGTTAACT

TGTTTTCATTTTGGTGGTTACCATCATGAACATCATTTGCATCCACATGTTCCATGGTGGAGATTGCCAAGAACAAGAA
AAACTGGTGGTAGAGCT

SspCrtW_Dmut
ATGACAGTTGATCATGATGCTAGAATTTCTTTGTTGTTAGCAGCTGCAATTGGTGCTGCATGGTTAGCTATTCATGTTG
GTGCTATTGTTTGGTGGAGATGGTCTCCAGCAACTGCTGTTTTGGCTATTCCAGTTGTTTTGGTTCAAGCATGGTTAT
CTACAGGTTTGTTTATTGTTGCTCATGATTGTATGCATGGTTCTTTTGTTCCAGGTAGACCAGCTGTTAATAGAACAGT
TGGTACTTTATGTTTGGGTGCATACGCTGGTTTATCTTACGGTCAATTGCATCCAAAACATCATGCACATCATGATGCA
CCAGGTACTGCAGCTGATCCAGATTTTCATGCTGGTGCACCAAGATCAGCTTTGCCATGGTTTGCTAGATTTTTCACA
TCTTATTACACACATGGTCAAATTTTAAGAATTACTGCTGCAGCTGTTTTGTACATGTTATTGGGTGTTTCTTTGTTAAA
TATTGTTGTTTTCTGGGCTTTGCCAGCATTAATTGCTTTGGCACAATTGTTTGTTTTTGGTACATTTTTGCCACATAGAC
ATGGTGACACACCATTTGCTGATGCACATAATGCTAGATCAAATGGTTGGCCATGGTTGGCTTCTTTAGCAACTTGT
TTTCATTTGGGTGCTTATCATCATGAACATCATTTGTCTCCATGGACACCATGGTGGCAATTGCCAAGAGTTGGTCAA
CCAGCAGCTGGTCATAGATCATTGTCTAAAGATAGA

HpCrtZ
ATGTTGTCTAAATTGCAATCTATCTCAGTTAAGGCTAGAAGAGTTGAATTAGCAAGAGATATCACTAGACCAAAGGTTT
GTTTGCATGCTCAAAGATGTTCATTGGTTAGATTAAGAGTTGCTGCACCACAAACTGAAGAAGCTGTTGGTACACAAC
AAGCTGCAGGTGCAGGTGACGAACATTCTGCAGATGTTGCTTTGCAACAATTAGATAGAGCAATTGCTGAAAGAAGA
GCTAGAAGAAAGAGAGAACAATTGTCATACCAAGCTGCAGCTATTGCAGCTTCTATTGGTGTTTCAGGTATCGCAATC
TTCGCTACTTACTTGAGATTCGCTATGCATATGACAGTTGGTGGTGCTGTTCCATGGGGTGAAGTTGCAGGTACTTTG
TTATTGGTTGTTGGTGGTGCATTGGGTATGGAAATGTATGCAAGATACGCTCATAAAGCAATTTGGCATGAATCTCCA
TTGGGTTGGTTATTGCATAAATCACATCATACTCCAAGAACAGGTCCATTCGAAGCTAACGATTTGTTCGCAATTATTA
ACGGTTTGCCAGCTATGTTGTTGTGTACATTCGGTTTCTGGTTGCCAAATGTTTTAGGTACTGCTTGTTTTGGTGCAG
GTTTGGGTATCACATTGTACGGTATGGCCTACATGTTTGTTCATGATGGTTTAGTTCATAGAAGATTTCCAACTGGTCC
AATTGCTGGTTTGCCATACATGAAGAGATTGACAGTTGCACATCAATTGCATCATTCTGGTAAATACGGTGGTGCTCC
ATGGGGCATGTTTTTGGGTCCACAAGAATTACAACATATTCCAGGTGCAGCTGAAGAAGTTGAAAGATTAGTTTTGGA
ATTGGATTGGTCAAAAAGA

AaCrtZ
ATGGCAAATTTCTTGATCGTTGTTGCTACTGTTTTGGTTATGGAATTGACAGCATACTCTGTTCATAGATGGATTATGC
ATGGTCCATTGGGTTGGGGTTGGCATAAATCACATCATGAAGAACATGATCATGCTTTGGAAAAGAATGATTTGTACG
GTTTAGTTTTCGCTGTTATTGCA

ACTGTTTTGTTTACAGTTGGTTGGATTTGGGCACCAGTTTTGTGGTGGATTGCTTTGGGTATGACTGTTTACGGTTTGA
TCTATTTCGTTTTGCATGATGGTTTAGTTCATCAAAGATGGCCTTTTAGATACATCCCAAGAAAGGGTTACGCTAGAAG
ATTGTACCAAGCACATAGATTACATCATGCTGTTGAAGGTAGAGATCATTGTGTTTCTTTTGGTTTTATATATGCACCA
CCAGTTGATAAATTGAAGCAAGATTTGAAGATGTCAGGTGTTTTGAGAGCTGAAGCACAAGAAAGAACA

AspCrtZ
ATGACTCAATTCTTGATCGTTGTTGCTACTGTTTTGGTTATGGAATTGACAGCATACTCTGTTCATAGATGGATTATGC
ATGGTCCATTAGGTTGGGGTTGGCATAAATCACATCATGAAGAACATGATCATGCTTTGGAAAAGAATGATTTGTACG
GTGTTGTTTTCGCTGTTTTGGCAACTATCTTGTTTACTGTTGGTGCTTACTGGTGGCCAGTTTTGTGGTGGATTGCATT
GGGTATGACAGTTTACGGTTTGATCTATTTCATCTTGCATGATGGTTTAGTTCATCAAAGATGGCCTTTTAGATACATC
CCAAGAAGAGGTTACTTTAGAAGATTGTACCAAGCTCATAGATTACATCATGCAGTTGAAGGTAGAGATCATTGTGTTT
CTTTTGGTTTTATATATGCACCACCAGTTGATAAATTGAAGCAAGATTTGAAGAGATCTGGTGTTTTGAGACCACAAGA
TGAAAGACCATCA

PaCrtZ
ATGTTGTGGATCTGGAACGCTTTGATCGTTTTCGTTACTGTTATTGGTATGGAAGTTATTGCTGCATTGGCTCATAAGT
ACATCATGCATGGTTGGGGTTGGGGTTGGCATTTGTCTCATCATGAACCAAGAAAGGGTGCTTTCGAAGTTAACGATT
TGTACGCAGTTGTTTTCGCTGCATTGTCTATCTTGTTGATATATTTGGGTTCAACTGGCATGTGGCCATTACAATGGAT
TGGTGCTGGTATGACAGCATACGGTTTGTTGTACTTCATGGTTCATGATGGTTTGGTTCATCAAAGATGGCCTTTTAG
ATACATCCCAAGAAAGGGTTATTTGAAGAGATTGTACATGGCTCATAGAATGCATCATGCAGTTAGAGGTAAAGAAGG
TTGTGTTTCTTTTGGTTTCTTGTACGCTCCACCATTGTCAAAATTGCAAGCAACATTAAGAGAAAGACATGGTGCTAGA
GCAGGTGCTGCAAGAGATGCTCAAGGTGGTGAAGATGAACCAGCATCAGGTAAA

BrevCrtZ
ATGGCTTGGTTGACTTGGATCGCTTTGTTTTTAACAGCATTTTTAGGCATGGAAGCATTTGCATGGATCATGCATAGAT
ACGTTATGCATGGTTTCTTGTGGTCTTGGCATAGATCACATCATGAACCACATGATCATCCATTGGAAAAGAATGATTT
GTTCGCAGTTGTTTTCGCTGCACCAGCTATTGTTATGGTTGCAGTTGGTTTGCATTTGTGGCCATGGGCTTTGCCAGT
TGGTTTAGGTATCACTGCATACGGTATGGTTTACTTTTTCTTTCATGATGGTTTAGTTCATAGAAGATTCCCAACTGGTT
TTTCTGGTAGATCAGGTTTTTGGACAAGAAGAATACAAGCTCATAGATTGCATCATGCAGTTAGAACAAGAGAAGGTT
GTGTTTCTTTTGGTTTCTTGTGGGTTAGATCAGCTAGAGCATTGAAAGCTGAATTAGCACAAAAGAGAGGTTCTTCATC
TTCAGGTGCT

PmCrtZ

ATGACTAATTTCTTGATCGTTGTTGCAACTGTTTTGGTTATGGAATTGACAGCTTACTCTGTTCATAGATGGATTATGCA
TGGTCCATTGGGTTGGGGTTGGCATAAATCACATCATGAAGAACATGATCATGCTTTGGAAAAGAATGATTTGTACGG
TTTAGTTTTCGCTGTTATTGCAACTGTTTTGTTTACAGTTGGTTGGATTTGGGCACCAGTTTTGTGGTGGATTGCTTTG
GGTATGACAGTTTACGGTTTGATCTATTTCGTTTTGCATGATGGTTTAGTTCATCAAAGATGGCCTTTTAGATATGTTC
CAAGAAAAGGTTATGCTAGAAGATTGTACCAAGCACATAGATTACATCATGCTGTTGAAGGTAGAGATCATTGTGTTT
CTTTTGGTTTTATATATGCTCCACCAGTTGATAAATTGAAGCAAGATTTGAAGACTTCAGGTGTTTTGAGAGCTGAAGC
ACAAGAAAGAACA

PspCrtZ
ATGACTAATTTCTTGATCGTTGTTGCAACTGTTTTGGTTATGGAATTGACAGCTTACTCTGTTCATAGATGGATTATGCA
TGGTCCATTGGGTTGGGGTTGGCATAAATCACATCATGAAGAACATGATCATGCTTTGGAAAAGAATGATTTGTACGG
TTTAGTTTTCGCTGTTATTGCAACTGTTTTGTTTACAGTTGGTTGGATTTGGGCACCAGTTTTGTGGTGGATTGCTTTG
GGTATGACAGTTTACGGTTTGATCTATTTCGTTTTGCATGATGGTTTAGTTCATCAAAGATGGCCTTTTAGATACATCC
CAAGAAAGGGTTACGCTAGAAGATTGTACCAAGCACATAGATTACATCATGCTGTTGAAGGTAGAGATCATTGTGTTT
CTTTTGGTTTTATATATGCTCCACCAGTTGATAAATTGAAGCAAGATTTGAAGATGTCAGGTGTTTTGAGAGCTGAAGC
ACAAGAAAGAACT
